# Germinal Center T follicular helper (GC-Tfh) cell impairment in chronic HIV infection involves c-Maf signaling

**DOI:** 10.1371/journal.ppat.1009732

**Published:** 2021-07-19

**Authors:** Marita Chakhtoura, Mike Fang, Rafael Cubas, Margaret H. O’Connor, Carmen N. Nichols, Brian Richardson, Aarthi Talla, Susan Moir, Mark J. Cameron, Virginie Tardif, Elias K. Haddad

**Affiliations:** 1 Department of Medicine, Division of Infectious Diseases & HIV Medicine, Drexel University College of Medicine, Philadelphia, Pennsylvania, United States of America; 2 Department of Population and Quantitative Health Services, Case Western Reserve University, Cleveland, Ohio, United States of America; 3 Iovance Biotherapeutics, San Carlos, California, United States of America; 4 Department of Molecular and Cellular Biology and Genetics, Drexel University College of Medicine, Philadelphia, Pennsylvania, United States of America; 5 Allen Institute for Immunology, Seattle, Washington, United States of America; 6 Laboratory of Immunoregulation, National Institute of Allergy and Infectious Diseases, National Institutes of Health, Bethesda, Maryland, United States of America; 7 Sorbonne University, INSERM, Center of Reasearch in Myology (Association Institut de Myologie) UMRS 974, AP-HP, Department of Internal Medicine and Clinical Immunology, DHU I2B, Pitié-Salpêtrière Hospital, Paris, France; 8 Department of Microbiology and Immunology, Drexel University College of Medicine, Philadelphia, Pennsylvania, United States of America; Emory University, UNITED STATES

## Abstract

We have recently demonstrated that the function of T follicular helper (Tfh) cells from lymph nodes (LN) of HIV-infected individuals is impaired. We found that these cells were unable to provide proper help to germinal center (GC)-B cells, as observed by altered and inefficient anti-HIV antibody response and premature death of memory B cells. The underlying molecular mechanisms of this dysfunction remain poorly defined. Herein, we have used a unique transcriptional approach to identify these molecular defects. We consequently determined the transcriptional profiles of LN GC-Tfh cells following their interactions with LN GC-B cells from HIV-infected and HIV-uninfected individuals, rather than analyzing resting *ex-vivo* GC-Tfh cells. We observed that proliferating GC-Tfh cells from HIV-infected subjects were transcriptionally different than their HIV-uninfected counterparts, and displayed a significant downregulation of immune- and GC-Tfh-associated pathways and genes. Our results strongly demonstrated that *MAF* (coding for the transcription factor c-Maf) and its upstream signaling pathway mediators (*IL6R and STAT3*) were significantly downregulated in HIV-infected subjects, which could contribute to the impaired GC-Tfh and GC-B cell functions reported during infection. We further showed that c-Maf function was associated with the adenosine pathway and that the signaling upstream c-Maf could be partially restored by adenosine deaminase -1 (ADA-1) supplementation. Overall, we identified a novel mechanism that contributes to GC-Tfh cell impairment during HIV infection. Understanding how GC-Tfh cell function is altered in HIV is crucial and could provide critical information about the mechanisms leading to the development and maintenance of effective anti-HIV antibodies.

## Author summary

Human immunodeficiency virus (HIV) remains a worldwide burden despite available treatments. The virus induces dysregulations in major immune cells and organs including lymph nodes. Germinal center T follicular helper (GC-Tfh) cells are immune cells which induce specific anti-HIV antibodies by helping GC-B cells. In chronic HIV, the interaction between these two cell types is defective, leading to modified and inefficient anti-HIV antibody responses. In this study, we examined the underlying mechanisms of this dysfunction. We observed that proliferating GC-Tfh cells from HIV-infected individuals, displayed distinctive gene expression than those from -uninfected subjects, following GC-B cell interaction. Furthermore, GC-Tfh cells from HIV patients showed a reduction in important immune-related pathway and gene expression. A number of essential GC-Tfh cell genes, such as *MAF* and its associated genes (*IL6R* and *STAT3*), were particularly attenuated in HIV, contributing to the impaired cells function. Moreover, we found an association between *MAF* function and the key enzyme adenosine deaminase-1 (ADA-1), where supplementation with ADA-1 partially restored the dysfunctional signaling in GC-Tfh cells during chronic infection. Understanding how GC-Tfh cells are altered in HIV is critical to elucidate the mechanisms leading to effective anti-HIV antibodies.

## Introduction

Germinal center T follicular helper (GC-Tfh) cells are CD4^+^ T cells present in secondary lymphoid organs and are key for the induction and maintenance of the humoral immune response [1–4]. They function in providing help to GC-B cells by playing a crucial role in affinity maturation and somatic hypermutation [5–7]. The differentiation of GC-Tfh cells is multifactorial and mainly influenced by IL-6 [2,8–10], inducible costimulator (ICOS) [11–13], IL-2 [13–15], the T cell receptor (TCR) [16], V-maf musculoaponeurotic fibrosarcoma (c-Maf) [17,18] and B cell lymphoma-6 (Bcl-6) [2,18,19]. Interestingly, the role of IL-6 in human GC-Tfh cell differentiation is not very clear, as IL-6 has been recently shown to have little or no effect on the differentiation of human GC-Tfh cells from CD4^+^ T cells *in vitro*, contrary to the case in mice [13,20]. Nevertheless, IL-6 remains one of the most important cytokines enhancing GC-Tfh cell function [21–23]. Bcl-6 is the key GC-Tfh transcription factor, which inhibits the expression of other T helper cell subset transcription factors [10,17,24–26], and is particularly necessary for the cells expression of CXCR5, which enables them to enter the GC [2,9,27]. GC-Tfh cells express characteristic markers of the helper program and are thus CXCR5^hi^ PD-1^hi^ Bcl-6^hi^ Maf^hi^ [18,28–30]. Alterations in GC-Tfh cells and their function have been observed in multiple settings, with a severe impact on the immune response. In HIV, the dysregulation observed in secondary lymphoid organs encompassed the inability of GC-Tfh cells to provide proper help to GC-B cells and consequently altered the efficacy of anti-HIV antibodies and elicited premature death of memory B cells [31].

c-Maf is a proto-oncogene which also acts as a crucial transcription factor heavily involved in the differentiation [18], maintenance [32], survival [33,34] and function [18,32] of GC-Tfh cells. Importantly, c-Maf is a significant contributor to IL-21 [32,33,35] and IL-4 [18,36] cytokine production by GC-Tfh cells, necessary to induce GC-B cell proliferation [18,37–39]. In mice, c-Maf knockout in the T cell compartment led to the absence of expression of the key GC-Tfh cell markers Bcl-6, CXCR5 and PD-1, as well as a reduction in high-affinity antibody production [17]. In humans, transduction of tonsillar CD4^+^ T cells with a maf-expressing lentivirus, induced GC-Tfh-associated gene expression (CXCR5, CXCR4 and PD-1) in naïve cells, as well as IL-21 secretion by non-Tfh, pre-Tfh and GC-Tfh cells [18], highlighting c-Maf’s importance for GC-Tfh cell development and function. c-Maf is expressed downstream of ICOS and BATF, which both regulate its expression [17]. Furthermore, the pro-GC-Tfh cytokine IL-6 as well as the transcription factors STAT3 and BATF upstream of c-Maf, are all essential mediators of its signaling in GC-Tfh cells [10,36,40–42].

Human immunodeficiency virus (HIV) infects millions of people worldwide, with global morbidity and mortality despite the remarkable progress in available treatments. In secondary lymphoid organs, sites where the anti-HIV immune response is mounted, the virus has been shown to induce a predominant dysregulation and disturbance of the microenvironment [43–45]. In chronically HIV-infected individuals, impaired GC-Tfh cells were demonstrated to provide inadequate help to GC-B cells, resulting in an altered and inefficient humoral response, recapitulated as well in Simian Immunodeficiency Virus (SIV)-positive rhesus macaques [31]. The defect was shown to be partially due to the enhanced interaction of PD-1 on GC-Tfh with PD-L1 on GC-B cell surfaces. This interaction resulted in decreased cell proliferation, activation, ICOS expression as well as IL-21 production, and was rescued by PD-1 signaling blockade or IL-21 supplementation [31]. However, a complete understanding of the underlying mechanisms remains to be elucidated. Studies investigating GC-Tfh cell function have recently demonstrated the association of these cells as well as their peripheral blood counterparts, circulating (cTfh) cells, with the generation of protective broadly neutralizing antibodies (bNAbs) against HIV, Simian-HIV (SHIV) and SIV in humans and non-human primates (NHP) respectively [43,46–50]. Passive immunization with bNAbs has been shown to induce transient, but efficient antiviral activity in humans [51–53] and NHP [54–58]. These findings provide a proof of concept that HIV vaccines that are able to generate such antibodies might protect against HIV infection. The discovery of bNAbs in a number of HIV-infected individuals/rhesus macaques, revealed and emphasized the potency and importance of GC-Tfh and cTfh cells in mounting a strong immune response against the virus, with the ability to control the infection [43,46–50]. Unfortunately, current HIV vaccine approaches have failed to elicit such protective antibody responses. Thus, understanding the impairment of GC-Tfh cells in chronic HIV-infected subjects becomes critical and informative of the mechanisms leading to the development and maintenance of effective anti-HIV antibodies.

Adenosine deaminase-1 (ADA-1) is a ubiquitously expressed key enzyme of the purine salvage metabolism pathway, with highest levels observed in lymphoid tissues [59–61]. It is expressed intracellularly but is also cell surface-bound, and possesses enzymatic as well as non-enzymatic functions [21,61]. ADA-1 is responsible for the irreversible deamination of adenosine and 2’deoxyadenosine into inosine and 2’deoxyinosine, and therefore the absence or impairment of ADA-1 function results in extra and intracellular accumulation of toxic adenosine, 2’deoxyadenosine and deoxyadenosine triphosphate (dATP) [21,61]. Absence or impairment in ADA-1 is additionally associated with the development of severe combined immunodeficiency (SCID) in humans, where no T, B or NK cells are present [59,62], but also non-immunologic manifestations including liver, skeletal, cognitive and behavioral abnormalities are observed [59]. ADA-1 binds four G-protein coupled receptors (GPCR) for adenosine A_1_R, A_2A_R, A_2B_R and A_3_R, and activates them to mediate its effects [59,63,64]. The non-enzymatic effects of ADA-1 include effects on the immune system [21,65], such as costimulation of T cell activation, by enhancing the bridging of T cells with dendritic cells [65]. We have recently described a novel function of ADA-1 showing that it plays a critical role in enhancing GC-Tfh cell differentiation and function [66]. ADA-1 expression was almost undetectable in GC-Tfh cells from HIV-infected subjects, and could thus be involved in these cells dysfunction [21].

The underlying molecular mechanisms of GC-Tfh cell dysfunction in chronic HIV remain poorly defined. In this paper, we used a unique gene array to identify the molecular defects. We determined for the first time, the transcriptional profiles of HIV^pos^ and HIV^neg^ lymph node (LN) GC-Tfh cells, following their interaction with GC-B cells. We observed that proliferating HIV^pos^ and HIV^neg^ GC-Tfh cells were transcriptionally distinct and that *MAF* (encoding the transcription factor c-Maf) and its upstream signaling pathway mediators (*IL6R and STAT3*) were significantly downregulated in HIV^pos^ cells, which contributes to the impaired GC-Tfh and GC-B cell interaction. We further showed the association of c-Maf function with the adenosine pathway and that the expression of the IL-6 pathway in HIV^pos^ GC-Tfh cells could be restored by ADA-1 supplementation, partially rescuing the dysregulation identified in GC-Tfh cells.

## Results

### LN GC-Tfh cells from HIV^pos^ individuals exhibit inadequate helper function despite uncompromised proliferation ability

**Fig 1:**
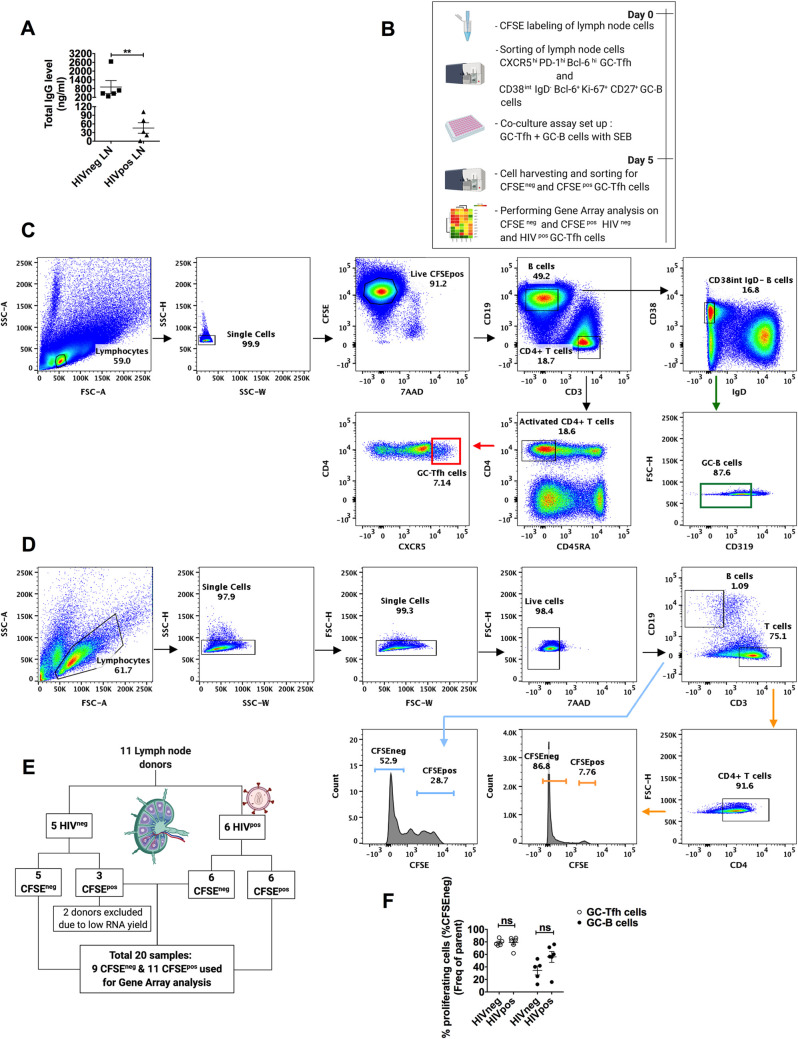
Experimental design used for gene array analysis. **(A) shows the total IgG expression levels in supernatants from autologous germinal center (GC)-Tfh and GC-B cell co-cultures from HIV negative (HIV**^**neg**^**) and HIV positive (HIV**^**pos**^**) lymph nodes (LNs).** Supernatants were collected on day 5 of the co-culture and analyzed for total IgG levels by ELISA. Closed squares (n = 5) depict HIV^neg^ and closed triangles (n = 5) depict HIV^pos^ individuals. Results are shown in ng/ml and are represented as mean ± SEM. Data was analyzed with the unpaired two-tailed Student’s t-test using the Mann-Whitney test. Nominal p-values p<0.05 were considered statistically significant. ** p<0.01. **(B) shows the co-culture/sorting strategy used for gene array analysis.** At day 0, LN mononuclear cells from HIV^neg^ and HIV^pos^ individuals were labeled with CFSE, sorted into GC-Tfh and GC-B cells and co-cultured in the presence of Staphylococcal Enterotoxin B (SEB). At day 5, each cell population was re-sorted according to their CFSE expression (proliferating = CFSE^neg^ or non-proliferating = CFSE^pos^) and prepared for gene array analysis. **(C) Representative conventional flow cytometry plots showing hierarchal phenotype gating strategy of sorted CFSE-labeled LN GC-Tfh and GC-B cell populations from HIV**^**neg**^ **or HIV**^**pos**^ **individuals** used in the co-culture assay. At day 0, sorted GC-Tfh cells were CFSE^+^ 7AAD^-^ CD19^-^ CD3^+^ CD4^+^ CD45RA^-^ CXCR5^hi^. Sorted GC-B cells were CFSE^+^ 7AAD^-^ CD3^-^ CD19^+^ CD38^int^ IgD^-^ CD319^-^. **(D) Representative flow cytometry plots showing hierarchal phenotype gating strategy of the re-sorted GC-Tfh and GC-B cells after 5 days of co-culture, based on their CFSE expression levels.** 7AAD^-^ CD3^+^ CD4^+^ GC-Tfh as well as 7AAD^-^ CD19^+^ GC-B cells were sorted into CFSE^neg^ and CFSE^pos^ cells for gene array analysis. **(E) shows the scheme used in the gene array assay.** LNs from 11 donors (n = 5 HIV^neg^ versus n = 6 HIV^pos^) were included in this experiment. Cells were labeled with CFSE, sorted, co-cultured and re-sorted for gene array analysis, as described above. Due to quality control, analyzed samples consisted of n = 8 HIV^neg^ (5 CFSE^neg^ and 3 CFSE^pos^) and n **=** 12 HIV^pos^ (6 CFSE^neg^ and 6 CFSE^pos^) cell populations. **(F) shows the percent of proliferating (CFSE**^**neg**^**) GC-Tfh (open circles) and GC-B (closed circles) cells from HIV**^**neg**^ **subjects (n = 5) and HIV**^**pos**^ **patients (n = 6), 5 days after co-culture.** Results are represented as mean ± SEM. Data was analyzed using one-way ANOVA followed by the Tukey multiple comparisons test. Nominal p-values p<0.05 were considered of statistical significance. ns indicates absence of statistical significance. **Fig 1B** and **1E** were created with BioRender.com.

HIV induces a predominant dysregulation of the microenvironment in secondary lymphoid organs, affecting GC activities, including cellular and humoral immunity [31,43]. A hallmark of this impaired immunity is in the inability to generate an effective and protective humoral response. We measured the function of GC-Tfh cells from LNs of HIV-infected patients and compared it to those from HIV-uninfected individuals, using an *in vitro* co-culture assay with autologous GC-B cells, as previously reported [43,47,67,68]. We observed a significant decrease in total IgG production, 5 days after co-culture of GC-Tfh cells with GC-B cells from LNs of HIV^pos^ individuals as compared to HIV-uninfected subjects (**Fig 1A and [31]**). We have previously attributed this defect, at least in part, to PD-1/PD-L1 interactions on GC-Tfh and GC-B cells respectively. However, the primary players driving this dysfunction are not yet fully identified. To study these mechanisms, sorted CFSE-labeled GC-Tfh cells from HIV^pos^ and HIV^neg^ LNs were co-cultured with autologous GC-B cells in the presence of Staphylococcal Enterotoxin B (SEB), as described in Materials and Methods and **Fig 1B.** The representative cell sorting gating strategy prior to co-culture is shown in **Fig 1C**. GC-Tfh cells were CXCR5^hi^ (**Fig 1C**) as well as PD-1^hi^ and Bcl-6^hi^ [31], while GC-B cells were CD38^int^, IgD^-^, CD319^-^ (**Fig 1C**), CD27^+^ [69], Bcl-6^+^ and Ki-67^+^ [70]. After 5 days of co-culture, we re-sorted the cells based on their proliferation status using CFSE staining (CFSE^neg^ = proliferating and CFSE^pos^ = non-proliferating), following the representative gating strategy depicted in **Fig 1D**. The resulting sample population, shown in **Fig 1E,** was used in gene array analysis. We detected no significant differences in the frequency of proliferating (CFSE^neg^) GC-Tfh and GC-B cells from healthy versus HIV^pos^ individuals by flow cytometric analysis (**Fig 1F)**. This indicates that the dysfunction in GC-Tfh cells from infected individuals is not due to their inability to proliferate, but more likely to their inability to efficiently interact with GC-B cells and consequently provide them with adequate help.

### Gene array analysis reveals distinct transcriptional profiles of GC-Tfh cells following their interactions with GC-B cells

**Fig 2:**
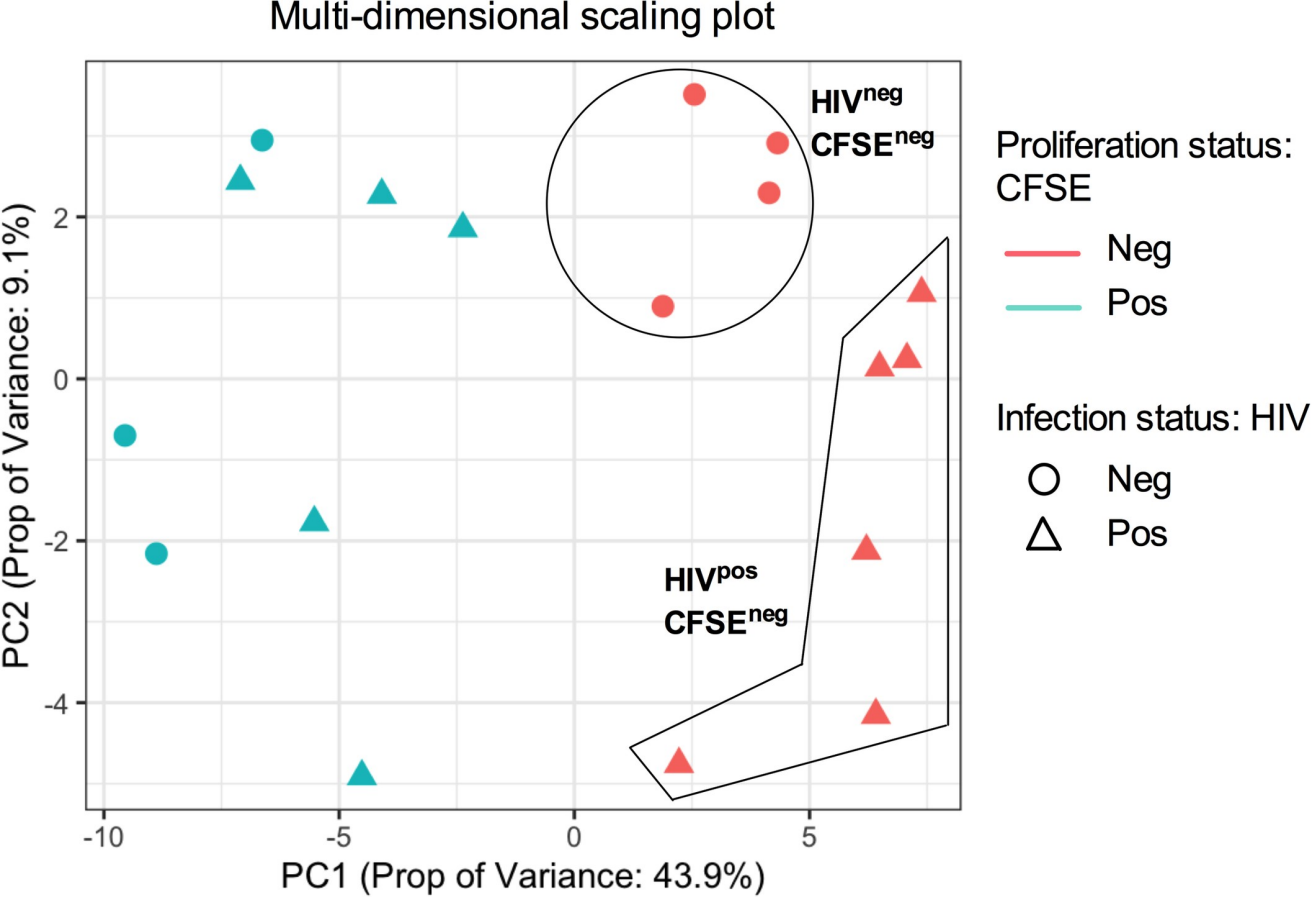
Multi-dimensional Scaling Plot (MDS) reveals unique proliferation- and infection-related gene profile clustering in GC-Tfh cells from HIV^pos^ versus HIV^neg^ individuals. We performed single-gene analysis of divided/activated versus non-proliferating LN GC-Tfh cells after 5 days of co-culture with autologous GC-B cells from HIV^pos^ patients versus healthy subjects. Principle component (PC) analysis shown in the MDS plot along the first and second principle components, provides a visual representation of the similarity or distance between datasets from all samples. GC-Tfh cells were analyzed based on whether they are proliferating (CFSE negative CFSE^neg^: pink data points) or non-proliferating (CFSE positive CFSE^pos^: green data points), but also based on infection status (HIV negative HIV^neg^: circles or HIV positive HIV^pos^: triangles). MDS plot shows gene expression clustering between proliferating versus non-proliferating GC-Tfh cells as well as between proliferating HIV^pos^ versus HIV^neg^ GC-Tfh cells. Each symbol represents one sample. CFSE^neg^ HIV^neg^: n = 4; CFSE^neg^ HIV^pos^: n = 6; CFSE^pos^ HIV^neg^: n = 3; CFSE^pos^ HIV^pos^: n = 5).

We used gene array analysis to identify the underlying mechanisms of GC-Tfh cell impairment during chronic HIV infection. **Fig 2** represents a multi-dimensional scale (MDS) analysis, displaying gene clustering of the different groups. MDS plot analysis provides a visual representation of the similarity or distance between the datasets from each sample, and is shown here along the first two principle components (PC). Our MDS analysis revealed apparent clustering of proliferating (CFSE^neg^ [pink data points]) versus non-proliferating (CFSE^pos^ [green data points]) LN GC-Tfh cells. Interestingly and within dividing cells, we observed clustering of GC-Tfh cells from HIV-infected (HIV^pos^ [pink triangles]) versus -uninfected (HIV^neg^ [pink circles]) individuals (**Fig 2**). These results revealed the important observations that the transcriptome of proliferating GC-Tfh cells is distinct in comparison with their non-dividing counterparts and that proliferating GC-Tfh cells from HIV^pos^ LNs are different compared to those from HIV^neg^ LNs.

### Differential gene expression highlights dividing LN GC-Tfh cells from HIV-infected and HIV-uninfected individuals

**Fig 3:**
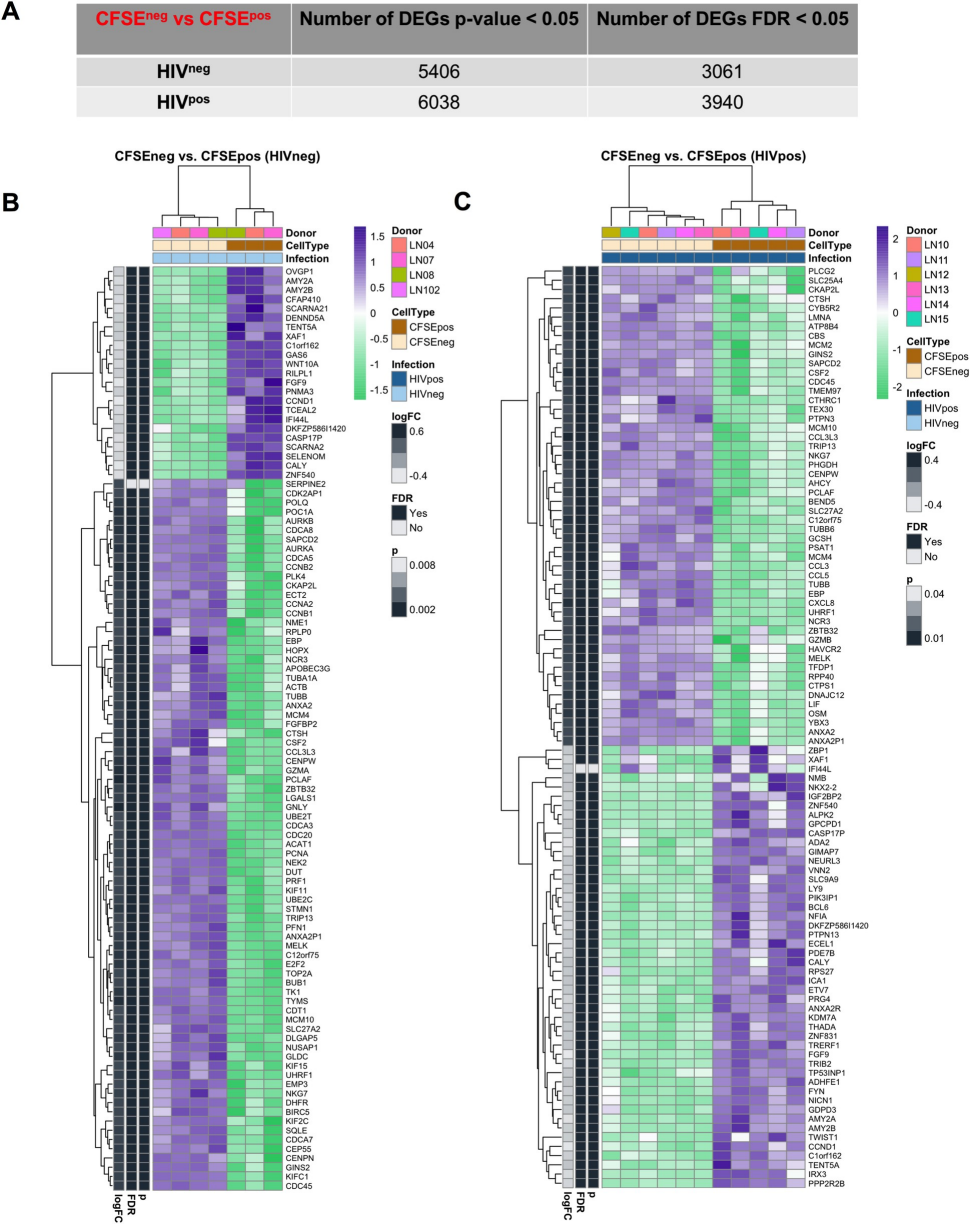
Differential gene expression characterizes proliferating GC-Tfh cells from LNs of HIV-infected and HIV-uninfected individuals. We used single-gene analysis to determine the pattern of gene expression in proliferating LN GC-Tfh cells in co-culture with autologous GC-B cells from HIV^pos^ patients versus HIV^neg^ subjects. **(A) Table summarizing differential gene expression analysis.** Table shows the number of differentially expressed genes (DEGs) specific to proliferating (CFSE^neg^ vs CFSE^pos^) HIV^neg^ and HIV^pos^ GC-Tfh cells after 5 days of co-culture with GC-B cells, selected for analysis based on nominal p-value p<0.05 or Benjamini Hochberg adjusted p-value (false discovery rate (FDR)) of <0.05. **(B-C) Heatmaps of the top 100 DEGs in proliferating GC-Tfh cells from (B) HIV**^**neg**^ **and (C) HIV**^**pos**^ **individuals.** We generated the heatmaps of the top 100 DEGs based on the highest absolute log2-fold change (logFC) in proliferating (CFSE^neg^ vs CFSE^pos^) GC-Tfh cells from HIV-uninfected and HIV-infected subjects compared to non-dividing cells. Nominal and Benjamini Hochberg adjusted p-values (or FDR) of <0.05 were considered statistically significant. Upregulated genes are highlighted in purple and downregulated genes are highlighted in green.

We used single-gene analysis to determine the gene expression pattern of proliferating LN GC-Tfh cells following their co-culture with autologous GC-B cells from HIV-infected and HIV-uninfected individuals. Our results showed a large number of differentially expressed genes (DEGs) in proliferating HIV^pos^ and HIV^neg^ GC-Tfh cells compared to non-dividing cells (**Fig 3**). The number of DEGs is summarized based on the rigor of the analysis. Proliferating GC-Tfh cells in co-culture with GC-B cells from HIV^neg^ LNs, displayed a total of 5406 DEGs based on a significant nominal p-value (p<0.05) and 3061 DEGs based on a Benjamini Hochberg adjusted p-value or false discovery rate (FDR<0.05) versus a total of 6038 and 3940 DEGs respectively for HIV^pos^ proliferating GC-Tfh cells (**Fig 3A**).

We subsequently generated separate heatmaps of the top 100 DEGs in dividing GC-Tfh cells from HIV^neg^ and HIV^pos^ individuals (**Fig 3B and 3C and S1 Table)**. Out of the top 100 DEGs in proliferating HIV^neg^ and HIV^pos^ LN GC-Tfh cells, we observed as expected, upregulation in genes consistent with processes required for or leading to cell activation and proliferation, with many of these DEGs being commonly upregulated in cells from infected individuals and their healthy counterparts. Among these, were genes involved in DNA replication (*MCM4*, *GINS2*, *CDC45*, *MCM10*), mitosis (*CKAP2L CENPW*) and cell cycle regulation (*MELK*), DNA damage and repair (*MCM10*, *TRIP13*, *PCLAF*, *UHRF1*), chemotaxis (*CCL3L3*), regulation of T helper cell differentiation and activation (*ZBTB32*) as well as metabolism (*SLC27A2*, *EBP*) (**Fig 3B and 3C**). On the other hand, following GC-Tfh: GC-B cell co-culture, proliferating HIV^neg^ GC-Tfh cells displayed a downregulation in the expression of multiple DEGs, many of which were also attenuated in HIV^pos^ GC-Tfh cells, such as those involved in transcription repression (*ZNF540*), regulation of cell proliferation, differentiation and migration (*FGF9*) as well as pro-apoptotic factors (*XAF1*) (**Fig 3B and 3C).** These findings emphasize the importance of these genes and their related pathways/processes in both HIV^neg^ and HIV^pos^ GC-Tfh cells, and highlight the ability of the two cell populations to undergo cell maintenance, regulation, activation and proliferation. In addition, we detected the upregulation of multiple HIV-inhibiting DEGs, mostly relevant in the cells from HIV-infected individuals. In fact, the increased expression of *CCL3L3*, *CCL3* and *CCL5* in proliferating HIV^pos^ GC-Tfh cells, constitutes a possible mechanism for the cells to control the virus (**Fig 3C**). Moreover, we observed DEG downregulation, specific to HIV^pos^ GC-Tfh cells (**Fig 3C)**. Essentially, we found a reduction in the expression of *ZBP1*, a gene with a role in host defense against pathogens, acting as a cytoplasmic DNA sensor and inducing the IFN-I response [71]. This attenuation may indicate a modulation of the immune response by HIV. Furthermore, we detected in the top 100 DEG list of proliferating HIV^pos^ GC-Tfh cells, a decrease in the expression of *ADA2*, coding for an enzyme involved in the adenosine pathway and playing a role in the regulation of cell proliferation and differentiation [64] (**Fig 3C)**. Interestingly, the master GC-Tfh cell transcription factor *BCL6* was also in the top 100 downregulated DEGs in proliferating HIV^pos^ GC-Tfh cells (**Fig 3C)**. Taken together, this data underlines the ability of dividing HIV^pos^ GC-Tfh cells to undergo vital cell processes including cell activation and proliferation, and highlights the large number of specific DEG alterations, induced by HIV.

### Proliferating GC-Tfh cells from HIV^neg^ and HIV^pos^ LNs show enrichment of metabolic, DNA repair and cell cycle regulation pathways

**Fig 4:**
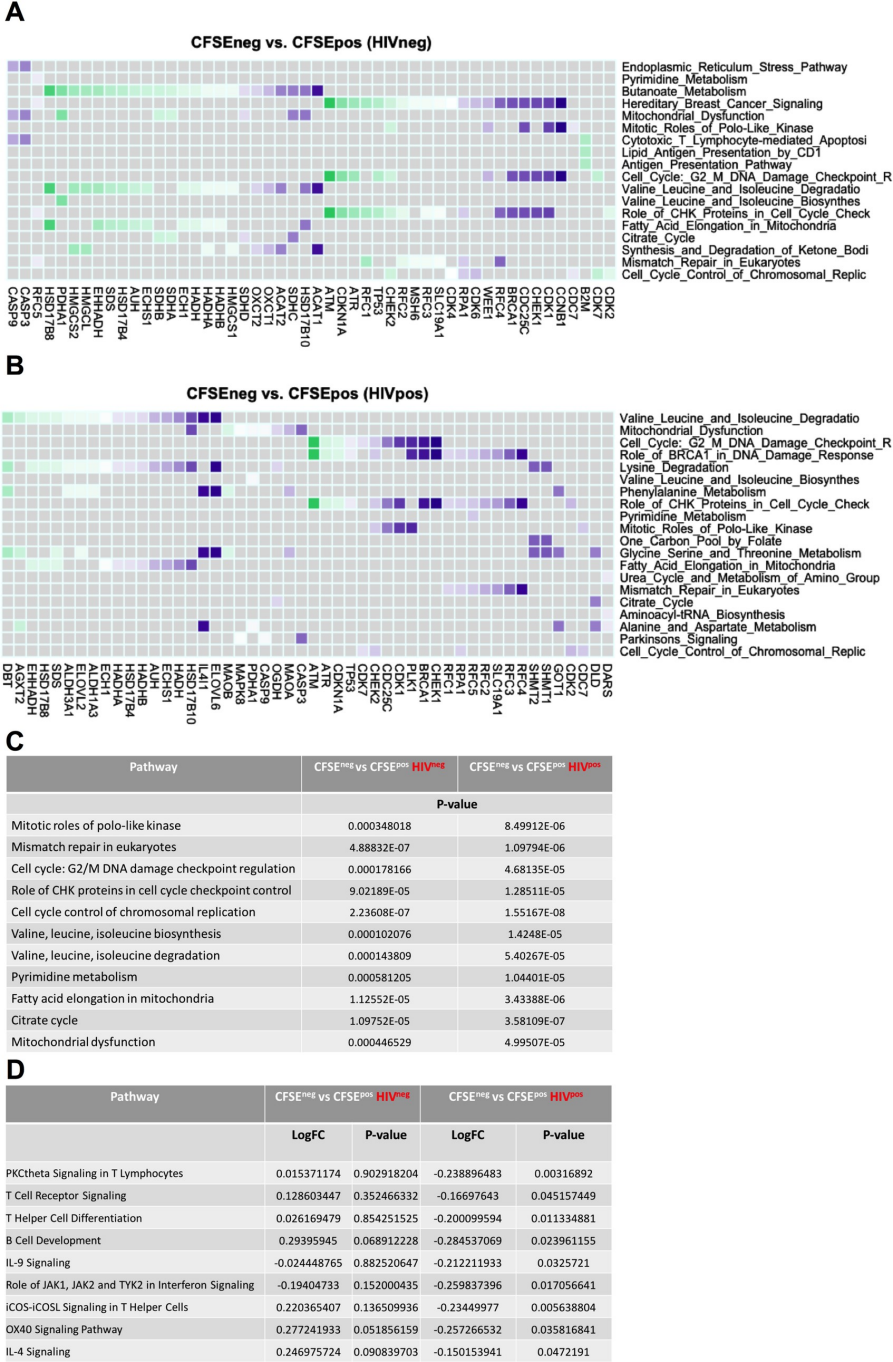
Pathway analysis reveals an alteration of immune-related pathway expression in proliferating HIV^pos^ GC-Tfh cells. Gene Set Variation Analysis (GSVA) was used to determine the biological characterization, statistical significance and differences in selected databases. We performed enrichment analysis using Igenuity Pathway Analysis (IPA) gene sets to determine the profile of GC-Tfh cell proliferation in the context of HIV or a non-HIV environment. **(A-B) Checkerboard plots represent the top list of enriched pathways in the DEGs specific to proliferating GC-Tfh cells in the (A) HIV**^**neg**^ **and (B) HIV**^**pos**^ **contexts. Commonly enriched cell cycle signaling and regulation, DNA repair and metabolism pathways are significantly enriched in both settings, at p<0.05.** The genes on the x axes in **A** and **B** are drivers of the specific pathway enrichments on the y axes. Genes shown in the heatmaps have the highest positive and negative log2-fold change (logFC), while pathways within the heatmaps are the top nominally significant based on GSVA enrichment analysis at p<0.05. Genes highlighted in purple are upregulated and genes highlighted in green are downregulated. **(C) Table showing the commonly enriched pathways in the top pathway list between proliferating (CFSE**^**neg**^ **vs CFSE**^**pos**^**) HIV**^**neg**^ **and HIV**^**pos**^ **GC-Tfh cells.** Nominal p-values p<0.05 were considered significant. **(D) Table showing a selection of enriched GC-Tfh-associated immunological pathways, altered in proliferating (CFSE**^**neg**^ **vs CFSE**^**pos**^**) HIV**^**pos**^ **versus HIV**^**neg**^ **cells based on analysis by IPA.** Statistical significance was considered with nominal p-values p<0.05.

To further explore the transcriptome of dividing GC-Tfh cells, we performed pathway exploration using Gene Set Variation Analysis **(**GSVA) in HIV-infected versus -uninfected subjects. We compared differential pathways expressed in proliferating GC-Tfh cells between HIV^neg^ and HIV^pos^ individuals using Ingenuity Pathway Analysis (IPA) defined gene sets. We examined the top list of enriched pathways in the DEGs specific to proliferating GC-Tfh cells in the HIV^pos^ versus HIV^neg^ populations (**Fig 4A–4C and S2 Table)**. As expected, we observed a predominance for metabolism, DNA repair and cell cycle regulation pathways enriched in both HIV^pos^ and HIV^neg^ settings (**Fig 4A and 4B**). Specifically, 11 pathways encompassing cell cycle signaling and regulation, DNA repair as well as metabolism, were commonly enriched and upregulated in both proliferating HIV^neg^ and HIV^pos^ GC-Tfh cells, as shown in **Fig 4A and 4B and S2 Table)**. The mitotic roles of polo-like kinase (p<0.001 for HIV^neg^; p<0.0001 for HIV^pos^) was the commonly enriched cell cycle signaling pathway, the mismatch repair in eukaryotes (p<0.0001 for HIV^neg^ and HIV^pos^) was the commonly enriched DNA repair pathway, while cell cycle regulation pathways included the cell cycle: G2/M DNA damage checkpoint regulation (p<0.001 for HIV^neg^; p<0.0001 for HIV^pos^), the role of CHK proteins in cell cycle checkpoint control (p<0.0001 for HIV^neg^ and HIV^pos^) and the cell cycle control of chromosomal replication (p<0.0001 for HIV^neg^ and HIV^pos^) (**Fig 4A–4C and S2 Table)**. This finding emphasizes the importance of these pathways for the regulated proliferation of both HIV^neg^ and HIV^pos^ GC-Tfh cells and for being key for this process to occur. In fact, the enrichment of the mitotic roles of polo-like kinase pathway is consistent with the activation of mitosis, necessary for cell triggering and proliferation [72] of the two cell populations. The mismatch repair in eukaryotes pathway is consistent with DNA replication fidelity and correction of mutations needed for maintenance of proliferating HIV^pos^ and HIV^neg^ GC-Tfh cells and their viability [73]. Furthermore, the enrichment of the cell cycle checkpoint pathways, is consistent with the cells crucial temporary ability in both GC-Tfh populations, to halt the cell cycle, allowing for repair of DNA damage or completion of DNA replication [74]. Moreover, 6 of the 11 commonly enriched pathways between proliferating HIV^pos^ and HIV^neg^ GC-Tfh cells were metabolism pathways, which included the valine, leucine, isoleucine biosynthesis (p<0.001 for HIV^neg^; p<0.0001 for HIV^pos^) as well as degradation pathways (p<0.001 for HIV^neg^; p<0.0001 for HIV^pos^), the pyrimidine metabolism (p<0.001 for HIV^neg^; p<0.0001 for HIV^pos^), the fatty acid elongation in mitochondria (p<0.0001 for HIV^neg^ and HIV^pos^), the citrate cycle (p<0.0001 for HIV^neg^ and HIV^pos^) and the mitochondrial dysfunction (p<0.001 for HIV^neg^; p<0.0001 for HIV^pos^) pathways (**Fig 4A–4C and S2 Table**). These pathways encompassed reactions/cycles essential for cell survival, respiration, activation, proliferation and recycling of intermediates, which, based on our data, are necessary and functional in both GC-Tfh cell populations (**Fig 4A and 4B**). These data demonstrate a differential gene expression predominant in cell cycle, repair and metabolism pathways of proliferating HIV^neg^ and HIV^pos^ GC-Tfh cells and highlight the significance of the enriched pathways to both cell populations, independent of infection.

### Proliferating GC-Tfh cells from HIV^pos^ LNs display a downregulation in key GC-Tfh cell-associated immunological pathways

Further analysis into immune-associated pathways revealed the alteration of a significant number of these in dividing GC-Tfh cells from HIV-infected patients, compared to those from uninfected subjects. We initially investigated pathways that are associated with GC immune functions and compared their expression/enrichment between proliferating (CFSE^neg^ vs CFSE^pos^) HIV^pos^ and HIV^neg^ populations (**Fig 4D)**. Interestingly, proliferating HIV^pos^ GC-Tfh cells exhibited a significant downregulation in all the selected enriched immunological pathways, indicating a mutli-level immune dysregulation. We first observed a general alteration, at the T lymphocyte signaling level, as dictated by the downregulation of the enriched PKCtheta signaling in T lymphocytes (p<0.01), as well as the T cell receptor signaling (p<0.05) pathways (**Fig 4D).** We also detected a specific and significant attenuation of the T helper cell differentiation pathway (p<0.05), which directly affects GC-Tfh cell development due to their helper phenotype, but also consequently pathways of B cell development (p<0.05) and IL-9 signaling (p<0.05) (**Fig 4D).** The latter two pathways are rather closely interconnected and somewhat GC-Tfh cell-dependent [75]. Moreover, we observed a downregulation in the role of Jak1, Jak2 and Tyk2 in interferon signaling pathway (p<0.05) in proliferating HIV^pos^ GC-Tfh cells, which may directly affect c-Maf and thereby GC-Tfh cell signaling, since Jak1, Jak2 and Tyk2 are all involved in the IL-6/STAT3 signaling [76], upstream of c-Maf. Notably, GC-Tfh cell-associated pathways including ICOS-ICOSL signaling in T helper cells (p<0.01), OX40 (p<0.05) as well as IL-4 signaling (p<0.05) pathways were all significantly downregulated in dividing HIV^pos^ GC-Tfh cells (**Fig 4D**), emphasizing the defect in the interaction between GC-Tfh and GC-B cells in HIV. On the other hand, we observed positive enrichment of the OX40 signaling pathway in proliferating HIV^neg^ GC-Tfh cells, which was upregulated with a p-value closely approaching significance (p = 0.0518) (**Fig 4D).** Taken together, our findings strongly indicate robust dysregulations in immunological pathways, particularly key GC-Tfh cell-related immune pathways, which could possibly contribute to the impaired proliferating GC-Tfh cell interactions with GC B cells in HIV.

### c-Maf signaling is dysregulated in proliferating HIV^pos^ GC-Tfh cells

**Fig 5:**
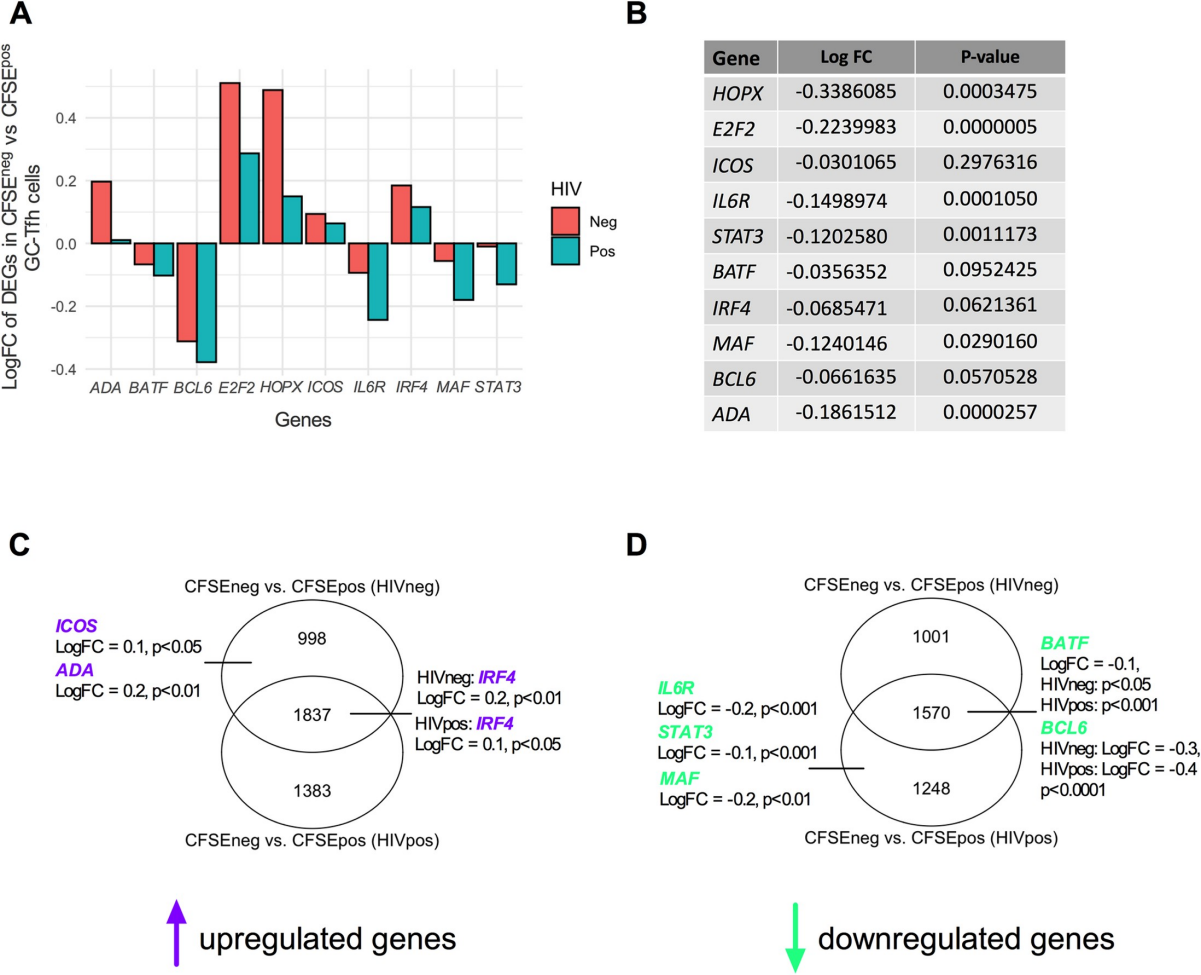
c-Maf signaling represents a key dysregulated pathway in proliferating HIV^pos^ GC-Tfh cells. **(A) shows log2-fold change (logFC) in the expression of selected DEGs in different cell signaling and GC-Tfh-associated immunological pathways in proliferating (CFSE**^**neg**^ **vs CFSE**^**pos**^**) HIV**^**neg**^ **and HIV**^**pos**^ **GC-Tfh cells.** Among these, are genes coding for transcription factors (*E2F2*) and coregulators (*HOPX*), enzymes (*ADA*) and key GC-Tfh genes (*ICOS*, *IRF4*, *BCL6*, *MAF* and mediators of its signaling pathway *IL6R*, *STAT3*, *BATF*). **(B) table showing log2-fold change (logFC) values and statistical significance between proliferating HIV**^**pos**^ **versus HIV**^**neg**^ **GC-Tfh cells for each selected DEG in (A).** To assess statistical significance between the logFC of proliferating HIV^pos^ versus HIV^neg^ GC-Tfh cells, data in (A) was analyzed using a double contrast. CFSE^neg^ values were baselined to CFSE^pos^ values and an HIV^pos^ versus HIV^neg^ contrast was performed to compare significance between (CFSE^neg^ vs CFSE^pos^) HIV^pos^ and (CFSE^neg^ vs CFSE^pos^) HIV^neg^ cells. **(C-D) Venn diagram analysis of proliferating (CFSE**^**neg**^ **vs CFSE**^**pos**^**) HIV**^**neg**^ **and HIV**^**pos**^ **GC-Tfh cells.** Diagrams show the numbers of unique and common statistically significant DEGs in the indicated GC-Tfh populations. **(C) shows Venn diagram analysis of upregulated DEGs** (represented in purple) and **(D) shows Venn diagram analysis of downregulated DEGs** (represented in green) in HIV^neg^ and HIV^pos^ cells. **(B-D)** Analysis was performed based on nominal p-value p<0.05.

We then assessed differentially expressed genes that could directly impact the function and survival of GC-Tfh cells, including *IL6R*, *STAT3*, *BATF*, *IRF4*, *MAF* and *BCL6* (**Fig 5A**). We also assessed the profiles of other DEGs such as the transcriptional coregulator *HOPX* and the transcription factor *E2F2*, as these factors play a critical role in CD4^+^ T helper cell function [77,78]. To be able to calculate statistical differences between CFSE^neg^ vs CFSE^pos^ (HIV^neg^) and CFSE^neg^ vs CFSE^pos^ (HIV^pos^) DEGs, we baselined CFSE^neg^ to CFSE^pos^ log2-Fold Change (logFC) values and performed an HIV^pos^ versus HIV^neg^ contrast referred to as a double contrast. We then performed statistical analysis on the difference in DEG expression between the two populations and considered p-values p<0.05 to be significant (**Fig 5B**). Of the analyzed DEGs, we found that *HOPX*, *E2F2* as well as the GC-Tfh cell-associated *MAF* and upstream mediators of its signaling (*IL6R and STAT3*) showed significant downregulation between proliferating HIV^pos^ versus HIV^neg^ GC-Tfh cells (p <0.001 for *HOPX*; p<0.0001 for *E2F2*; p<0.05 for *MAF*; p<0.001 for *IL6R* and p<0.01 for *STAT3*) (**Fig 5A and 5B**). c-Maf, encoded by *MAF*, is a transcription factor responsible for GC-Tfh cell differentiation/development [18,40], survival [33,34] and performance/function [18,32,40]. Other important GC-Tfh cell-associated DEGs, including *IRF4* (p<0.062), *BCL6* (p<0.057) and *BATF* (p<0.095), showed apparent but not significant blunted expression levels between HIV^pos^ versus HIV^neg^ proliferating GC-Tfh cells (**Fig 5A and 5B**). Thus, these data highlight a key role for *MAF* and its signaling, which could contribute to the underlying mechanisms behind the inadequate GC-Tfh cell function in HIV. It is worth mentioning that dividing GC-Tfh cells from uninfected subjects showed a downregulation in *IL6R*, *STAT3*, *BATF*, *MAF* and *BCL6* expression (**Fig 5A**), which may constitute a normal response after activation. However, the expression of those genes was primarily significantly decreased in dividing GC-Tfh cells from HIV^pos^ subjects (**Fig 5A and 5D**).

Moreover, ICOS has been shown to be important for GC-Tfh cell differentiation, activation and function in B cell help [79,80]. The ICOS/ICOSL axis is also an inducer of c-Maf in mice [81]. We observed that *ICOS* expression levels were decreased when comparing dividing HIV^pos^ to HIV^neg^ GC-Tfh cells, without however reaching statistical significance. This indicates that *ICOS* may not be a direct HIV target in those cells (**Fig 5A and 5B)**. Furthermore, we found that the gene coding for ADA-1 enzyme (*ADA*), was significantly upregulated in proliferating HIV^neg^ GC-Tfh cells (p<0.01), whereas its expression was robustly reduced in HIV (**Fig 5A and 5C and previously in [21]**), inducing a significant decrease when comparing the proliferating HIV^pos^ versus HIV^neg^ cells (p<0.0001) (**Fig 5A and 5B)**. ADA-1 is a ubiquitous enzyme involved in purine metabolism, with implications in immune health [61,82]. We have recently demonstrated that ADA-1 improves the quality of GC-Tfh cell function and enhances GC-Tfh cell differentiation [66] as well as delineates functions of cTfh sub-populations [21]. Taken together, these results underline a link between ADA-1 and GC-Tfh cells in the context of HIV.

Moreover, using Venn diagram analysis to further discern the differences between proliferating GC-Tfh cells in health and chronic HIV infection, we detected 998 DEGs that were uniquely upregulated in proliferating HIV^neg^ cells, 1383 DEGs uniquely upregulated in proliferating HIV^pos^ cells, whereas 1837 DEGs were upregulated in both GC-Tfh populations (**Fig 5C and S3 Table**). *ICOS* (p<0.05) and *ADA* (p<0.01) were detected among the 998 significantly upregulated genes in proliferating HIV^neg^ but not HIV^pos^ cells, while *IRF4* was among the 1837 genes significantly increased in both populations (p<0.01 for HIV^neg^ and p<0.05 for HIV^pos^) (**Fig 5C and S3 Table**). On the other hand, we detected 1001 uniquely downregulated DEGs in proliferating HIV^neg^ cells. We also observed that *IL6R* (p<0.001), *STAT3* (p<0.001) and *MAF* (p<0.01) were among the 1248 uniquely significantly downregulated genes in proliferating HIV^pos^ cells. *BATF* (p<0.05 for HIV^neg^ and p<0.001 for HIV^pos^) and *BCL6* (p<0.0001 for HIV^neg^ and HIV^pos^) however, were among the 1570 significantly downregulated DEGs in both cell types, with the decrease being greater in HIV^pos^ cells (**Fig 5D and S3 Table)**. Collectively, this data underlines the dysfunction in the ADA-1/IL-6/c-Maf signaling axis, consequently contributing to the impaired function of GC-Tfh cells in HIV.

### ADA-1 blockade reduces c-Maf expression in healthy tonsillar GC-Tfh cells in co-culture with GC-B cells

**Fig 6:**
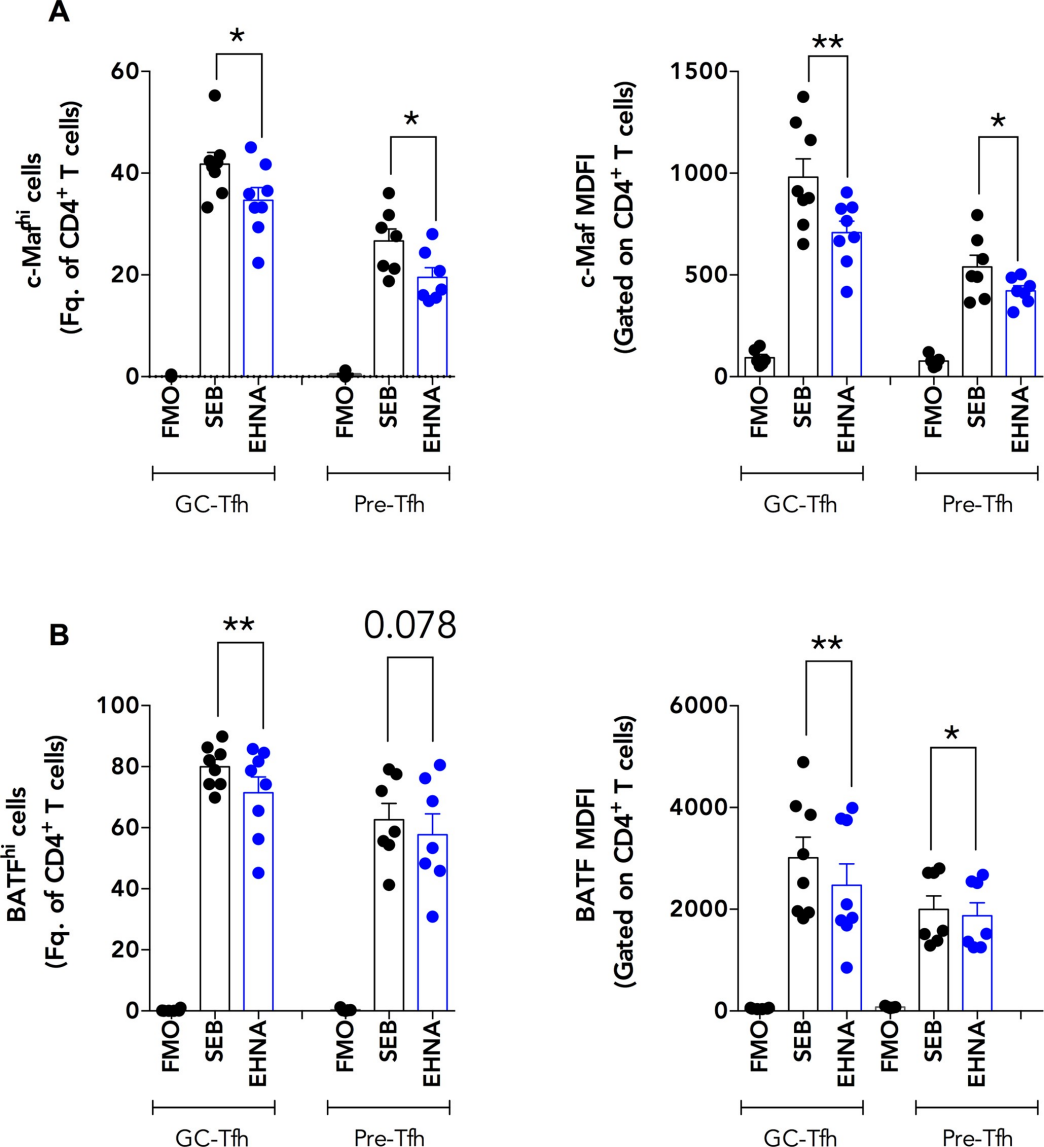
Specific ADA-1 blockade reduces c-Maf and BATF expression in healthy tonsillar GC-Tfh and pre-Tfh cells. We sorted GC-Tfh, pre-Tfh and autologous GC-B cells from the tonsils of healthy individuals and co-cultured GC-Tfh:GC-B as well as pre-Tfh:GC-B cells in a 1:1 ratio in the presence of SEB (SEB) with or without 10uM specific ADA-1 inhibitor EHNA (EHNA). We harvested the cells one day after co-culture and performed intracellular flow cytometry staining for c-Maf and BATF expression in GC-Tfh and pre-Tfh cells. **(A) shows the percent of c-Maf**^**hi**^ **GC-Tfh and pre-Tfh cells** in the total CD4^+^ T cell population **(left) and c-Maf median fluorescence intensity (MDFI) in GC-Tfh and pre-Tfh cells**, gated on the total CD4^+^ T cells **(right)** (n = 7–8). **(B) shows the percent of BATF**^**hi**^ **GC-Tfh and pre-Tfh cells** in the total CD4^+^ T cell population **(left) and BATF median fluorescence intensity (MDFI) in GC-Tfh and pre-Tfh cells**, gated on the total CD4^+^ T cells **(right)** (n = 7–8). Negative controls are labeled as Fluorescence Minus One (FMO). Results are from 3 independent experiments and are represented as mean ± SEM. Data was analyzed with the two-tailed paired non-parametric Student’s t-test using the Wilcoxon matched-pairs signed rank test. Nominal p-values p<0.05 were considered of statistical significance. * p<0.05 and ** p<0.01.

**Fig 7:**
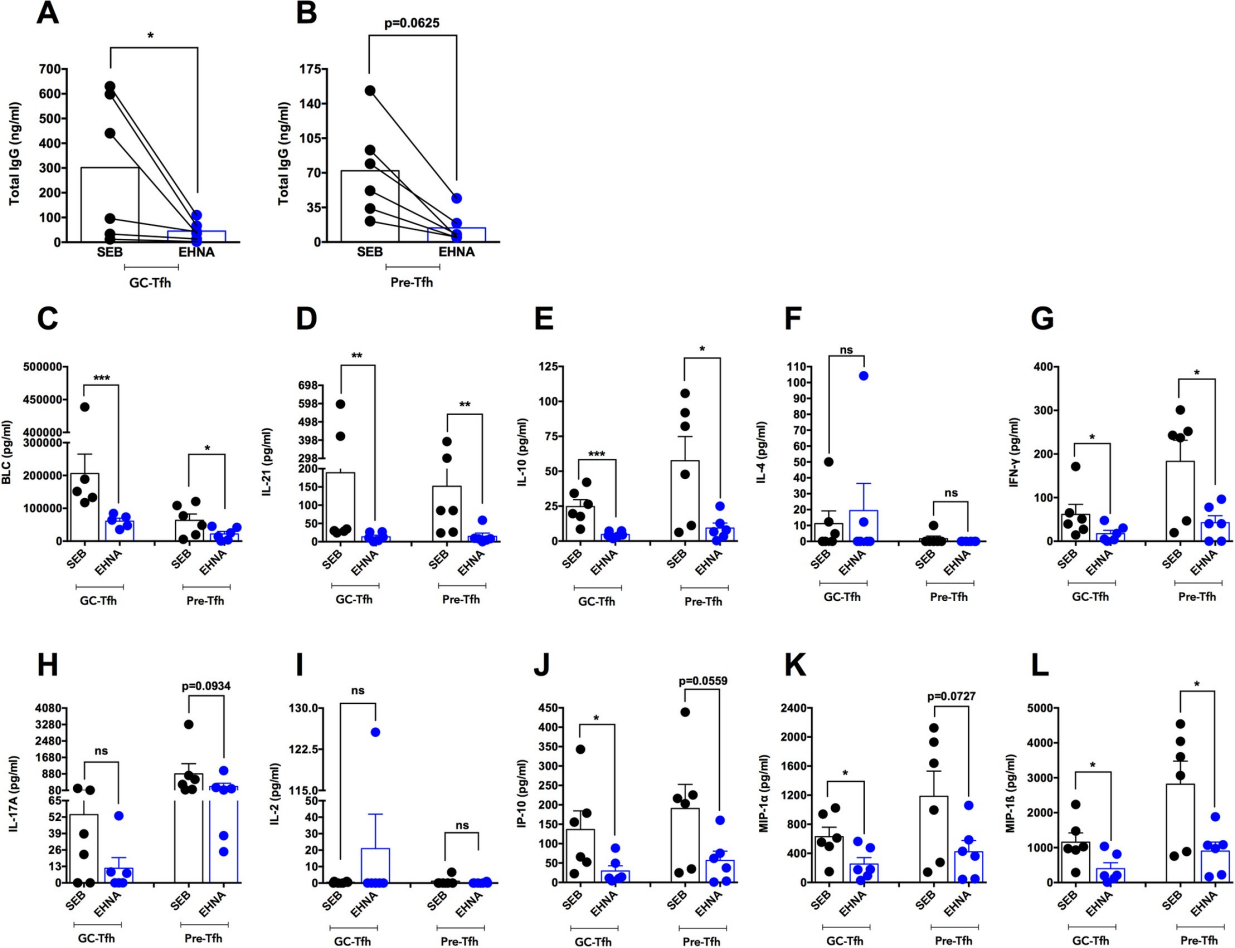
Specific ADA-1 blockade attenuates healthy tonsillar GC-Tfh cell function. We sorted GC-Tfh, pre-Tfh and autologous GC-B cells from the tonsils of healthy individuals and co-cultured GC-Tfh:GC-B as well as pre-Tfh:GC-B cells in a 1:1 ratio in the presence of SEB (SEB) with or without 10uM of the specific ADA-1 inhibitor EHNA (EHNA). **(A-B)** We collected culture supernatants 5 days after co-culture and analyzed the levels of total IgG in ng/ml by ELISA. **(A) Total IgG level in supernatant from GC-Tfh:GC-B cell co-cultures. (B) Total IgG level in supernatant from pre-Tfh:GC-B cell co-cultures.** Results are from 2 independent experiments (n = 6) and are represented as mean as well as before and after treatment lines. Data was analyzed with the two-tailed paired non-parametric Student’s t-test using the Wilcoxon matched-pairs signed rank test. **(C-L)** We collected culture supernatants 5 days after co-culture and analyzed GC-Tfh and pre-Tfh cell cytokine and chemokine levels by the Luminex assay. Levels of **(C) BLC, (D) IL-21, (E) IL-10, (F) IL-4, (G) IFN-γ, (H) IL-17A, (I) IL-2, (J) IP-10, (K) MIP-1α and (L) MIP-1ß** are shown in pg/ml. Results are from 2 independent experiments (n = 6) and are represented as mean ± SEM. Data was analyzed with the two-tailed unpaired non-parametric Student’s t-test using the Mann-Whitney test. Nominal p-values p<0.05 were considered statistically significant. * p<0.05, ** p<0.01 and *** p<0.001.

We previously showed that ADA-1 improved Tfh cell function *in vitro* [21]. Because our gene array findings demonstrated a dysregulation in the gene expression of *MAF* and its upstream signaling pathway (*IL6R*, *STAT3*) as well as in *ADA* in proliferating HIV^pos^ GC-Tfh cells (**Fig 5A and 5B**), we hypothesized that GC-Tfh cell impairment could be associated with the ADA-1/c-Maf axis. To test this hypothesis, we co-cultured sorted healthy human tonsillar GC-Tfh and pre-Tfh cells with GC-B cells as previously described [21,43], in the presence or absence of the ADA-1 specific inhibitor EHNA (erythro-9-(2-hydroxy-3-nonyl)adenine), which will inhibit the endogenous ADA-1 production. We stained harvested cells 1 day after co-culture to monitor intracellular c-Maf and BATF expression levels by flow cytometry. The gating strategy followed for the stained cells is illustrated in **S1 Fig**. We observed a significant decrease in the frequency of c-Maf-positive GC-Tfh and pre-Tfh cells (p<0.05), which correlated with c-Maf median fluorescence intensity (MDFI) in these cells (p<0.01 for GC Tfh and p<0.05 for pre-Tfh), upon ADA-1 inhibition as compared to the non-inhibited control (**Fig 6A**). Likewise, we detected a significant reduction in the percent and MDFI of BATF expression with EHNA in GC-Tfh cells (p<0.01) when compared to control (**Fig 6B**). BATF is a transcription factor upstream of c-Maf, responsible for c-Maf and Bcl-6 induction [17,41]. We also observed a slight decrease in BATF frequency in pre-Tfh cells in the presence of EHNA (p = 0.078), and a significant MDFI attenuation (p<0.05) as compared to control (**Fig 6B**). These findings were not due to an EHNA-mediated increase in cell death, as the percentages of live GC-Tfh and pre-Tfh cells were similar with and without EHNA (**S2 Fig**). Moreover, we measured total IgG levels in the GC-Tfh: GC-B and pre-Tfh: GC-B cell human tonsillar co-culture supernatants, 5 days after the start of the assay. Interestingly, with GC-Tfh cells, we observed a significant reduction of total IgG production by GC-B cells upon ADA-1 inhibition with EHNA as compared to control (p<0.05) (**Fig 7A**). We detected a similar trend with pre-Tfh cells, however, the decrease in IgG concentrations only approached statistical significance (p = 0.0625) (**Fig 7B).** Taken together, these results indicate that ADA-1 affects c-Maf expression and signaling in GC-Tfh and pre-Tfh cells. In addition, the defect observed at the gene level of ADA-1, c-Maf and its signaling pathway in proliferating HIV^pos^ GC-Tfh cells contributed, at least in part, to the dysregulated GC-Tfh/GC-B cell interaction. Subsequently, ADA-1 may directly affect GC-Tfh cell function by targeting c-Maf expression or its upstream signaling mediators.

### ADA-1 blockade attenuates cytokine/chemokine production by GC-Tfh cells in co-culture with GC-B cells

Since ADA-1 blockade reduced c-Maf and BATF expression in GC-Tfh and pre-Tfh cells from healthy human tonsils in co-culture with GC-B cells and attenuated IgG production, we tested whether it could affect GC-Tfh cell function by decreasing their ability to produce cytokines and chemokines. To investigate this hypothesis, we co-cultured sorted healthy tonsillar GC-Tfh and pre-Tfh cells with GC-B cells in the presence or absence of EHNA, and collected supernatants 5 days after co-culture. EHNA significantly attenuated the production of the key GC-Tfh-specific chemokines and cytokines BLC (CXCL13) (p<0.001), IL-21 (p<0.01) and IL-10 (p<0.001) by GC-Tfh cells as compared to control. We observed similar results with pre-Tfh cells (BLC p<0.05; IL-21 p<0.01 and IL-10 p<0.05) (**Fig 7C–7E**). This suggests that ADA-1 may affect GC-Tfh cell cytokine/chemokine secretion via targeting c-Maf and its upstream mediators. Furthermore, GC-Tfh cells have the ability to also secrete IFN-γ [13,18,24,83,84] and IL-17A [18,81]. In this study, GC-Tfh cells produced low levels of IFN-γ, which were significantly inhibited by EHNA (p<0.05), whereas pre-Tfh cells secreted higher IFN-γ concentrations, similarly attenuated by the ADA-1 inhibitor (p<0.05) (**Fig 7G**). The cells also produced IL-17A, however no change was detected with EHNA (**Fig 7H**). Moreover, we did not observe any significant production of IL-4 or IL-2 [21] by any of the T cell subsets in this set of experiments (**Fig 7F and 7I respectively**). Lastly, we found that GC-Tfh and pre-Tfh cells secreted a number of pro-inflammatory chemokines including IP-10, MIP-1α and MIP-1ß, which were either significantly decreased by EHNA or reduced to a level with a p-value approaching significance (IP-10: p<0.05 for GC-Tfh and p = 0.0559 for pre-Tfh; MIP-1α: p<0.05 for GC-Tfh and p = 0.0727 for pre-Tfh; MIP-1ß: p<0.05 for GC-Tfh and pre-Tfh) (**Fig 7J–7L respectively**). These results indicate that ADA-1 acts through c-Maf and BATF to induce GC-Tfh cell cytokine and chemokine production.

### ADA-1 supplementation in co-cultures from HIV^pos^ LNs rescues the dysregulation in mediators of the c-Maf signaling pathway in GC-Tfh cells

**Fig 8:**
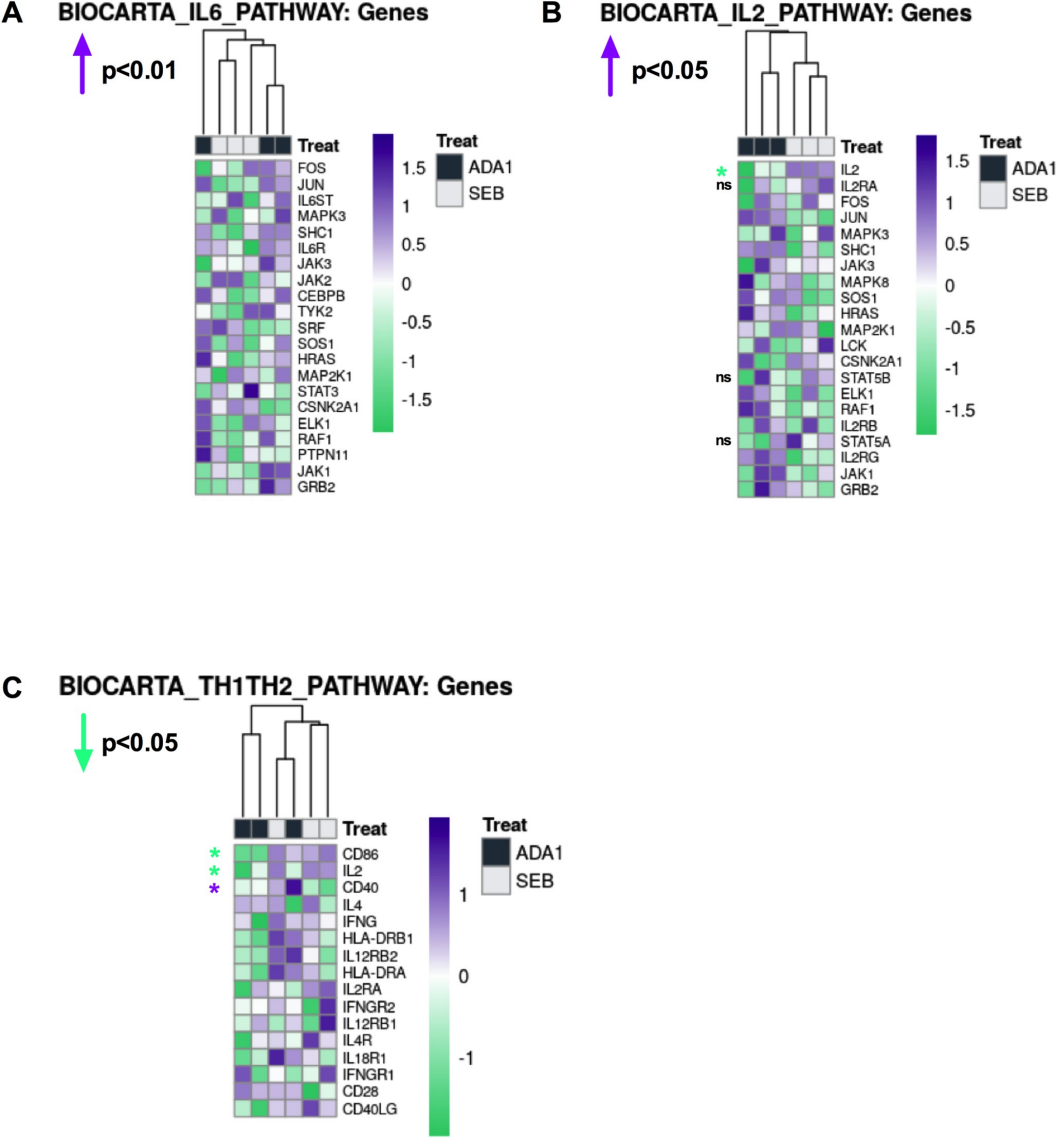
Co-culture supplementation with ADA-1 restores the defective pro-GC-Tfh IL-6 pathway in HIV^pos^ GC-Tfh cells. We sorted GC-Tfh and autologous GC-B cells from LNs of HIV^pos^ individuals with chronic infection and co-cultured them in a 1:1 ratio in the presence of SEB (SEB) with or without 2.4uM ADA-1 (ADA-1). Cells were harvested 1 day after co-culture then GC-Tfh cells were re-sorted and total RNA extracted for RNA-seq analysis. We analyzed the data using Gene Set Variation Analysis (GSVA) by examining Molecular Signatures Databases (MSigDB) Canonical Pathways. **Heatmaps illustrating the differential gene expression in the (A) IL-6, (B) IL-2 and (C) Th1/Th2 pathways in HIV**^**pos**^ **GC-Tfh cells treated in co-culture with ADA-1 versus SEB are represented.** RNA-seq analysis was performed on n = 3 SEB samples and n = 3 ADA-1 samples. Nominal p-values p<0.05 were considered statistically significant and are indicated for each pathway on the top left side. Where specified, statistical significance is shown for selected genes with *p<0.05. Absence of statistical significance for selected genes is represented by ns. Upregulated genes or pathways are highlighted in purple while downregulated genes or pathways are highlighted in green. Statistical analysis for all genes of the three pathways is shown in **S4 Table**.

Our results indicated that ADA-1 is a key player contributing to c-Maf expression as well as the proper function of GC-Tfh cells and their help to GC-B cells. We determined whether exogenous ADA-1 could restore the Maf signaling pathway by targeting c-Maf and/or upstream mediators of its signaling such as IL-6. To achieve this, we supplemented GC-Tfh: GC-B cell co-cultures from chronic HIV^pos^ LNs with ADA-1 and evaluated the transcriptional profile of GC-Tfh cells by RNA-seq analysis. We analyzed the data using Gene Set Variation Analysis (GSVA) by examining Molecular Signatures Databases (MSigDB) Canonical Pathways. We observed that ADA-1 induced a significantly robust overall upregulation of the IL-6 pathway (p<0.01) (**Fig 8A**), critical for c-Maf signaling and for healthy GC-Tfh cells and their function. This upregulation suggests a restoration of the IL-6 signaling, rescuing its prominent downregulation observed in HIV^pos^ GC-Tfh cells from co-cultures with SEB alone (**Figs 5A and 8A**). This is highly important, particularly since IL-6 is a pro-GC-Tfh cytokine and key mediator in the c-Maf signaling pathway. Moreover, in light with our previous report showing a correlation between an aberrant cTfh profile and an IL-2-responsive gene signature in chronic HIV [43], we examined the IL-2 pathway in our HIV^pos^ GC-Tfh cells and assessed the effect of co-culture supplementation with ADA-1 on its expression. Although the IL-2 pathway displayed an overall upregulation upon ADA-1 supplementation (p<0.05) (**Fig 8B**), its key DEG *IL2* was significantly downregulated with ADA-1 compared to the SEB control (p<0.05), and to a lesser extent without reaching significance, *IL2RA*, *STAT5A* and *STAT5B* (**Fig 8B and S4 Table**). This suggests that ADA-1 contributes to restoring, at least in part, the GC-Tfh phenotype and is reminiscent of the cTfh defect reversal in HIV upon interfering with the IL-2 signaling pathway [43]. Furthermore, consistent with the observed alterations in the IL-6 and IL-2 pathways in HIV^pos^ GC-Tfh cells with ADA-1, we identified an overall downregulation of the Th1/Th2 pathway (p<0.05) (**Fig 8C**). This was strongly illustrated by the sharp decrease in the expression of *IL2* (p<0.05), *CD86* (p<0.05) and increase in the expression of *CD40* (p<0.05) genes with ADA-1 versus control (**Fig 8C and S4 Table**). Taken together, our data suggest a partial rescue of the HIV^pos^ GC-Tfh cell dysfunction with ADA-1. Despite no direct effect of co-culture supplementation with ADA-1 on *MAF* expression, we identified the restoration of the IL-6 signaling pathway upstream of *MAF* and hallmark pro-GC-Tfh pathway, as well as the attenuation of the *IL2* gene expression and Th1/Th2 pathway.

## Discussion

Our previous study of the interactions between HIV^pos^ GC-Tfh and GC-B cells in co-culture, partially attributed the GC-Tfh cell defect to enhanced PD-1/PD-L1 interactions resulting in decreased ICOS expression, IL-21 secretion and IgG production [31]. Nevertheless, the mechanisms of this dysfunction are yet to be fully elucidated. Understanding how GC-Tfh cell function is altered in HIV is fundamental and could provide critical information about the mechanisms leading to the development and maintenance of effective anti-HIV antibodies.

In this study, we determined for the first time, the transcriptional profiles of HIV^pos^ and HIV^neg^ LN GC-Tfh cells, following their interaction with GC-B cells, and highlighted that *MAF* (encoding c-Maf) and its upstream signaling mediators (*IL6R*, *STAT3*), were altered in GC-Tfh cells from HIV-infected individuals. We further showed the association of c-Maf with the adenosine pathway and that ADA-1 supplementation could restore the expression of the IL-6 pathway, partially rescuing the dysregulation identified in HIV^pos^ GC-Tfh cells. Hence, this is the first report demonstrating a link between ADA-1, c-Maf and GC-Tfh cells in the context of HIV.

We examined GC-Tfh cell activation and function in chronic HIV infection, using the unique approach of studying their interaction with GC-B cells in co-culture, rather than in isolation *ex vivo* (**Fig 1B**). We showed for the first time, that the transcriptomes of proliferating and non-proliferating LN GC-Tfh cells were significantly different (**Fig 2**). This is not surprising as transcriptional profiles of dividing cells are different from those of non-dividing cells. More importantly, we observed that the transcriptional profiles of proliferating HIV^pos^ and HIV^neg^ GC-Tfh cells were distinct (**Fig 2**). This indicates that HIV alters the transcriptome of proliferating GC-Tfh cells following their interation with autologous GC-B cells. Despite the ability of GC-Tfh cells to proliferate comparably in both HIV-infected and -uninfected individuals (**Fig 1F**), HIV^pos^ GC-Tfh cells lost their ability to provide adequate help to GC-B cells. This was demonstrated by the sharp decrease in total IgG production following GC-Tfh: GC-B cell co-culture (**Fig 1A and [31])**. Hence, HIV alters proliferating GC-Tfh cell transcriptome by primarily targeting genes that are critical for the interaction with GC-B cells rather than those compromising their expansion and proliferation capacity. In fact, in chronic HIV infection, an expansion in the frequency of LN GC-Tfh cells has been reported in the literature [31,48,85] and ascribed to the chronicity of the infection and antigen accumulation [31,86,87]. In addition, when the cells were studied alone *in vitro*, non-interacting HIV^pos^ GC-Tfh cells, expressed similar levels of phenotypic markers (CXCR5, Bcl-6, PD-1, ICOS, CD40L) than their HIV^neg^ counterparts. Furthermore, their function was not impaired, and normal levels of IL-4, IL-10 and IL-21 were secreted, suggesting that the defect is not cell-intrinsic, but rather derives from HIV^pos^ GC-Tfh cell interaction with GC-B cells [31]. It is noteworthy to mention that GC-Tfh cells are also preferentially infected by HIV, where viral replication and virion production is significant [88–90]. In our previous studies [31,43], the inhibition of viral replication in GC-Tfh: GC-B cell co-culture, did not affect GC-Tfh cell function. Nonetheless, we believe it is possible that GC-Tfh cell infection with virus could also contribute to their dysfunction.

HIV altered a large number of DEGs in dividing LN GC-Tfh cells as compared to their non-dividing counterparts (**Fig 3A**). Yet, both proliferating HIV^pos^ and HIV^neg^ cells were able to undergo cell maintenance, regulation, activation and proliferation, upregulating and downregulating unique or common DEGs for the occurrence of these processes (**Fig 3B and 3C and S1 Table**). Interestingly, we observed the attenuation of the master GC-Tfh cell transcription factor in both proliferating HIV^neg^ and HIV^pos^ cells (p<0.0001) (**Fig 5A and 5D**). This is concurrent with the literature, which has demonstrated an attenuation of *BCL6* in healthy GC-Tfh cells after providing B cell help or to form memory [2,32,91,92], suggesting that this could reflect the downregulation normally occurring in this gene following B cell help, rather than indicate an HIV-induced alteration.

Interestingly however, the primary dysregulation in dividing HIV^pos^ GC-Tfh cells was detected in immunological and GC-Tfh cell-associated pathways, with a robust downregulation, as opposed to proliferating HIV^neg^ cells (**Fig 4D**). Alterations in the PKCtheta signaling in T lymphocytes and the T cell receptor signaling pathways, represented changes at the general level of T cell signaling, whereas alterations in the T helper cell differentiation pathway induced a helper-specific defect (**Fig 4D**), suggesting a direct impact on GC-Tfh cell development and function. Consequently, B cell development and IL-9 signaling pathways were likewise attenuated in proliferating GC-Tfh cells from HIV-infected individuals, since GC-Tfh cell-derived IL-9 production is known to impact GC development of memory B cells [75]. Furthermore, the downregulation of ICOS-ICOSL signaling in T helper cells, as well as OX40 and IL-4 signaling pathways (**Fig 4D**), indicates a GC-Tfh cell-specific dysfuntion. ICOS has been shown to act cooperatively with OX40 to amplify GC-Tfh cell development as well as GC reactions during infections [93]. Moreover, GC-Tfh cell production of IL-4, which is important for B cell help [13], is c-Maf-dependent [18,42], and a signaling pathway that is altered in HIV. Additionally, the attenuated role of Jak1, Jak2 and Tyk2 in interferon signaling pathway in HIV^pos^ GC-Tfh cells, could be the result of impaired IL-6/STAT3 signaling, which is part of the GC-Tfh cell transcription factor c-Maf pathway [10,36,40–42]. Although the IFN-I response is a major anti-viral mechanism and may confer some protection against infection [94,95], its upregulation, commonly found in cells from HIV-infected individuals, could also bear deleterious effects including disease progression, particularly if sustained [96,97]. On the other hand, HIV may block the IFN-I response and anti-viral gene expression, by disrupting STAT1 phosphorylation [98], degrading or reducing the phosphorylation of the JAK/STAT pathway components such as STAT1 and STAT3 [99]. Since STAT3 is a mediator of the IFN-I as well as the c-Maf signaling, thus, alterations in the IFN-I response may contribute to alterations in the c-Maf signaling observed in chronic HIV. Taken together, these data emphasize the dysregulation existing in proliferating HIV^pos^ GC-Tfh cells, impacting general and GC-Tfh cell-specific immune-related pathways and possibly contributing to the cells impaired interaction with GC-B cells.

Many DEGs were altered in proliferating HIV^pos^ as compared to HIV^neg^ GC-Tfh cells (**Fig 5**). The significant downregulation of *HOPX* and *E2F2* between proliferating HIV^pos^ versus HIV^neg^ GC-Tfh cells (**Fig 5A and 5B**) is interesting, given their importance in CD4^+^ T helper cell function. In fact, HOPX is expressed in human effector/memory Th1 cells and regulates the expression of apoptosis and survival genes in murine Th1 cells [77]. On the other hand, E2F2 acts as a transcriptional repressor of effector/memory CD4^+^ T cell proliferation in mice [78]. Furthermore, CXCR5^+^PD-1^+^CD8^+^ GC-Tfh cells upregulate E2F2 after murine LCMV infection [100]. Nevertheless, the role of HOPX and E2F2 in impacting the function of CD4^+^ GC-Tfh cells is important and remains to be elucidated. c-Maf is a key transcription factor downstream of IL-6, STAT3, BATF [10,36,40–42] and ICOS [17], and is essential for the development [18], maintenance and function [32] of GC-Tfh cells. Our finding by microarray analysis that *MAF*, the gene encoding c-Maf, as well as the upstream mediators of its signaling *IL6R* and *STAT3*, were significantly downregulated in dividing HIV^pos^ GC-Tfh cells (**Fig 5D**) but also when comparing the change in expression between proliferating HIV^pos^ and HIV^neg^ cells (**Fig 5A and 5B**), highlights the importance of *MAF* and its signaling for GC-Tfh cells. It additionally indicates their contribution to the impaired interaction of proliferating HIV^pos^ GC-Tfh with GC-B cells. Moreover, the downregulation in *IL6R*, *STAT3*, *BATF*, *MAF* and *BCL6* in dividing GC-Tfh cells from uninfected LNs (**Fig 5A)**, may constitute a normal response after activation. This has been reported for *BCL6*, when activated GC-Tfh cells are ready to exit the follicles towards other follicles or develop into memory Tfh cells [2,32,91,92]. On the other hand, the literature shows that upregulated *IRF4* can repress *BCL6* expression in healthy GC-B cells once the GC reaction ends, which renders *IRF4* upregulation consistent with *BCL6* attenuation [101]. Although this is concordant with our findings in proliferating HIV^neg^ GC-Tfh cells (**Fig 5A**), no other studies have investigated these dynamics in GC-Tfh cells. Furthermore, the significant upregulation in *ICOS* expression unique to healthy cells (**Fig 5C**), accompanied by a slight but non-significant downregulation in HIV (**Fig 5A and 5B**), suggests that *ICOS* may not be a direct HIV target in GC-Tfh cells. Indeed, Jurado et al showed that patients infected with HIV, did not display a change in T lymphocyte ICOS expression, contrary to those with tuberculosis co-infection, who exhibited ICOS upregulation [102].

Another important altered DEG was *ADA*, coding for the enzyme ADA-1. Alongside its enzymatic activity, ADA-1 is also involved in immune responses [61,65,82]. We have previously shown that ADA-1 promoted GC-Tfh cell differentiation [66] and improved the quality of the cells helper function [21]. Our finding of the significant upregulation in *ADA*, unique to dividing HIV^neg^ cells (**Fig 5C**) and significant downregulation when comparing expression between proliferating HIV^pos^ versus HIV^neg^ GC-Tfh cells (**Fig 5A and 5B**), confirms the important role of *ADA* in these cells, heavily impacted by the HIV-induced immune dysregulation. This is concurrent with the report of Martinez-Navio et al, showing that viral gp120-mediated disruption of ADA-CD26 interaction, is partially responsible for the immunological defects in T lymphocytes during HIV infection [103]. Likewise, we have previously shown the impairment of the ADA-CD26 axis in HIV, when comparing cTfh cells from virally suppressed patients versus elite controllers [21]. Nevertheless, the full mechanism of ADA-1 in GC-Tfh cells remains unclear.

Effectively, ADA-1 inhibition using the specific inhibitor EHNA, underlined the role of ADA-1 in healthy tonsillar GC-Tfh but also pre-Tfh cells and demonstrated a putative mechanism of action for its effects. The significant decrease in c-Maf and BATF expression upon ADA-1 blockade, indicated that the enzyme acts on GC- and pre-Tfh cells through the c-Maf signaling pathway (**Fig 6**). Furthermore, ADA-1 inhibition attenuated GC-Tfh cell function. In fact, the EHNA-mediated decrease in GC- and pre-Tfh cell chemokine and cytokine production, was mostly emphasized with the decrease in the GC-Tfh-specific BLC, IL-21 and IL-10 (**Fig 7C–7E**), consequently reducing the cells helper function, as observed with the reduction in IgG secretion (**Fig 7A and 7B**). The effects of EHNA were not due to compromised cell viability (**S2 Fig and [21])**. Essentially, we have previously demonstrated that EHNA does not affect the viability, proliferation or expression of surface key phenotypic markers of cTfh or memory B cells, despite reduction in IgG production in co-culture supernatant [21]. These results suggest that ADA-1 mediates the helper function of GC-Tfh cells via c-Maf and BATF. Moreover, c-Maf-induced IL-4 is produced by GC-Tfh cells to aid in their helper function. However, only a population but not all GC-Tfh cells do secrete this cytokine [13,18,42]. This could be an explanation for the absence of IL-4 in the SEB conditions of our ELISA (**Fig 7F**). Furthermore, IL-4 production by GC-Tfh cells may not be ADA-1-dependent at this late stage of the cells differentiation and helper function. Additionally, GC-Tfh cells have the capacity to secrete low levels of IFN-γ [18,24,83,84] and IL-17A [18,81]. The reduction of SEB-induced IFN-γ (**Fig 7G**) but not IL-17A (**Fig 7H**) concentrations with EHNA in both GC- and pre-Tfh cells, indicates that IL-17A production is independent of c-Maf and ADA-1 in these cells.

In chronic HIV, proliferating GC-Tfh cells displayed strong alterations in the expression of *ADA*, *MAF* and upstream mediators of its signaling, predominantly *IL6R* and *STAT3*, contributing to their inadequate interaction with GC-B cells. *IL6R* encodes the receptor to the pro-GC-Tfh cytokine IL-6, which is particularly essential for Bcl-6 regulation and c-Maf signaling [10,36,40–42], as well as for GC-Tfh cell differentiation [8–10,13,104] and response maintenance [105]. RNA-seq analysis showed that supplementing ADA-1 in HIV^pos^ GC-Tfh: GC-B cell co-cultures significantly upregulated, in GC-Tfh cells, the overall IL-6 pathway compared to control, and rescued the otherwise dysregulated IL-6 signaling (**Fig 8A**). *IL6R* and *IL6ST* DEGs were upregulated with ADA-1 versus SEB alone (**Fig 8A**), however, their upregulation did not reach statistical significance (**S4 Table**). The role of IL-6 in human GC-Tfh cell differentiation is not very clear, as IL-6 has been recently shown to have little or no effect on the differentiation of human GC-Tfh cells from CD4^+^ T cells *in vitro*, contrary to the case in mice [13,20]. Nevertheless, IL-6 remains one of the most important cytokines enhancing GC-Tfh cell function [21–23]. In addition, the downregulation in *STAT3* upon supplementation with ADA-1 versus SEB alone, could represent a normal response after activation, especially if the activation is transient. Furthermore, the downregulation of the *IL2* DEG with ADA-1 versus SEB alone despite the overall upregulation of the IL-2 pathway (**Fig 8B**), may constitute another evidence of GC-Tfh cell helper program rescue. Papillion et al have shown that the GC-Tfh cell response maintenance occurs with intrinsic IL-6 signaling and IL-2 hyporesponsiveness [105]. Our finding of *IL2* attenuation with ADA-1 versus SEB alone, may be due to its antagonism by IL-6 signaling, to a threshold which retains sufficient IL-2-mediated TCR signaling while preserving the GC-Tfh phenotype. Last but not least, the downregulation of the Th1/Th2 expression pathway post-ADA-1 supplementation in the co-culture (**Fig 8C**), indicates the predominance/maintenance of the GC-Tfh profile at the expense of the other helper profiles. Although we did not observe a rescue in *MAF* expression with ADA-1 supplementation, the upregulation of the IL-6 pathway upstream of *MAF*, represents a partial restoration of the c-Maf signaling in the GC-Tfh cells. The absence of direct restoration of *MAF* expression following ADA-1 exposure, could possibly be attributed to its unphased kinetics. Another interpretation may suggest a stronger control by ADA-1 on the IL-6/IL-2 axis, whereas c-Maf could be more strongly controlled by PD-L1-induced signaling through defective HIV^pos^ GC-B cells (**S4 Fig**). This suggests that upregulation of the upstream IL-6 pathway is not sufficient for effective restoration of the c-Maf pathway in HIV^pos^ GC-Tfh cells. Hence, it would be interesting to assess the effect of blocking the PD-1/PD-L1 interaction observed in HIV, on c-Maf, BATF and ADA-1 expression on the one hand, and the IL-6/IL-2 pathway on the other hand. Consequently, investigating whether ADA-1 supplementation along with PD-L1 blockade could fully restore GC-Tfh/GC-B cell interactions, would be crucial to fully decipher IL-6/c-Maf/ADA-1/PD-1/PD-L1 interconnections. We have previously published that ADA-1 does not induce antibody production from GC-B cells in isolation [21]. This demonstrates that ADA-1 supplementation in the HIV co-culture targets the GC-Tfh cells directly. Taken together, these findings highlight for the first time a link between ADA-1, GC-Tfh cells and the c-Maf signaling pathway in the context of HIV. These results indicate a putative role for ADA-1 in the rescue of the GC-Tfh cell program, in chronic HIV infection. It remains to be demonstrated if these ADA-1-induced effects translate into improving the GC-Tfh cell helper function and the anti-HIV humoral response.

In conclusion, we have shown for the first time using a unique gene array, that the transcriptional profiles of proliferating HIV^pos^ and HIV^neg^ LN GC-Tfh cells, are distinct. We also observed that dividing HIV^pos^ GC-Tfh cells displayed a dysregulation in multiple immune-related pathways. Interestingly, *MAF* (coding for c-Maf) and its upstream signaling pathway mediators (*IL6R* and *STAT3*) were altered in cells from infected individuals, which may contribute to the impaired GC-Tfh cell interaction with GC-B cells in chronic HIV. Moreover, we underlined the role of ADA-1 in the GC-Tfh cell function via c-Maf and BATF, and showed that ADA-1 may partially rescue the impairment in HIV^pos^ GC-Tfh cells by restoring the IL-6 signaling pathway, attenuating *IL2* and the Th1/Th2 pathway. Whether this ADA-1-mediated restoration may affect anti-HIV antibody production and rescue the inadequate helper function of GC-Tfh cells in chronic infection, warrants further investigation. Consequently, these observations would be of capital importance to unravel the mechanisms leading to the development and maintenance of effective anti-HIV antibodies.

## Materials and methods

### Ethics statement

Protocols for the collection of HIV^neg^ LNs as well as tonsils (TSLs) from healthy donors were all approved by the Institutional Review Board (IRB) of the Martin Memorial Health Systems in Stuart, Florida. Surgical biopsies of HIV^pos^ LNs were performed under protocols approved by the NIAID IRB (ClinicalTrials.gov Identifier: NCT00001316). All procedures were also approved by the IRB at Drexel University College of Medicine. All subjects signed a written informed consent before their participation.

### Human subjects, sample collection and processing

LNs from healthy and HIV-infected individuals were included in the study. A total of 5 HIV^neg^ and 8 HIV^pos^ LNs were collected (6 HIV^pos^ LNs were used in the microarray assay and 3 HIV^pos^ LNs were used in the RNA-seq assay. For one specimen, the same LN from one patient (different vials) was common to the 2 assays and for another specimen, 2 LNs from the same patient, collected at different time points, were used each in one assay). Mesenteric HIV^neg^ LNs were obtained from healthy individuals aged between 30 and 60 years old, admitted to the hospital for colectomy due to inflammation in the small intestine. These LNs were used as control since they are enriched in follicles and GC-Tfh cells in the absence of HIV infection. Surgical biopsies of palpable inguinal, cervical or axillary HIV^pos^ LNs were performed as previously indicated [106] at the National Institutes of Health Clinical Research Center in Bethesda, Maryland. All biopsies were from chronically infected, ART-naïve patients aged between 21 and 39 years old, with virus RNA plasma levels ranging between less than 50 and up to 293361 copies/ml and a CD4^+^ T cell count between 121 and 935 cells/ul. Excised LN samples were transported in RPMI at 4°C and then mechanically dispersed to obtain single cell suspensions as previously published [31].

TSLs from 9 healthy donors were obtained from the Martin Memorial Health Systems (Florida), processed as previously published [31], and the cells frozen until usage. The donors, aged between 20 and 45 years old, were undergoing routine tonsillectomy at the time of sample collection.

### CFSE-labeling of LN-derived HIV^neg^ and HIV^pos^ mononuclear cells

Cells were thawed in RPMI 1640 (Corning) supplemented with 10% fetal bovine serum (FBS) (Access Biologicals) and 1% penicillin/streptomycin (P/S) (Gibco), and resuspended at a density of 10x10^6^ cells/ml. They were then incubated with Benzonase (VWR) for 30 minutes at 37°C to remove any nucleic acid debris. The cells were subsequently washed twice in pre-warmed phosphate buffered saline (PBS) and resuspended in PBS at a maximum concentration of 20 x10^6^ cells/ml for staining with the Cell Trace CFSE Cell Proliferation kit (Life Technologies). CFSE was then added to the cell suspension at a 1.25uM concentration, and was incubated for 8 minutes after gentle mixing. Next, the CFSE staining was quenched with the addition of 5 volumes of ice-cold RPMI to the cells followed by a 5-minute incubation on ice. Cells were washed 3 times by centrifugation at 1500 rpm for 3 minutes and the pellet gently resuspended in fresh complete RPMI with HEPES (Thermo Fisher) after every wash. Cells were finally resuspended in the appropriate volume of sorting buffer.

### Cell sorting experiments

#### Sorting of CFSE-labeled *HIV*^*neg*^ *and HIV*^*pos*^ LN cells for gene array analysis

CFSE-labeled HIV^neg^ and HIV^pos^ LN mononuclear cells were resuspended in fluorescence activated cell sorting (FACS) buffer (RPMI without phenol red (Thermo Fisher) supplemented with 12.5mM HEPES, 10% FBS and 1% P/S) at a density of 50x10^6^ cells/ml for isolation of GC-Tfh and GC-B cells. Cells were incubated for 20 minutes at 4°C with fluorochrome-conjugated antibodies against the following human surface markers: CD3 (HIT3α), CD4 (RPA-T4), CD45RA (2H4LDH11LDB9), CXCR5 (RF8B2), CD19 (HIB19), CD38 (HIT2), IgD (IA6-2) and CD319 (162.1). All antibodies were purchased from Biolegend except for anti-CXCR5 and anti-CD45RA antibodies, which were purchased from BD Biosciences and Beckman Coulter respectively. Dead cells were excluded with the use of 7AAD (BD Biosciences). Cells were washed after incubation with 5ml buffer at 1200 rpm for 5 minutes and resuspended in FACS buffer at a concentration of 15x10^6^ cells/ml, filtered and sorted using a BD FACSAria II (BD Biosciences). After excluding doublets, sorted GC-Tfh cells were 7AAD^-^ CD3^+^ CD19^-^ CD4^+^ CD45RA^-^ CXCR5^hi^, while GC-B cells were 7AAD^-^ CD19^+^ CD3^-^ CD38^int^ IgD^-^ CD319^-^. Additionally, GC-Tfh cells were PD-1^hi^ as well as Bcl-6^hi^ [31] and GC-B cells Bcl-6^+^ Ki-67^+^ [70] and CD27^+^ [69]. All sorted cells were CFSE^pos^. The gating strategy followed for sorting of GC-Tfh and GC-B cells is shown in **Fig 1C**. Data was analyzed using FlowJo software (Treestar).

Five days after co-culture of the sorted CFSE-labeled GC-Tfh and autologous GC-B cells from HIV^neg^ and HIV^pos^ LNs, the cells were harvested and re-sorted as described above, based on their proliferation status. Proliferating cells were CFSE^neg^ while non-proliferating cells were CFSE^pos^. Antibodies against CD3, CD4 and CD19 were utilized. Dead cells were also excluded with the use of 7AAD. After eliminating doublets, proliferating GC-Tfh cells were 7AAD^-^ CD3^+^ CD19^-^ CD4^+^ CFSE^neg^ and non-dividing GC-Tfh cells were 7AAD^-^ CD3^+^ CD19^-^ CD4^+^ CFSE^pos^, whereas proliferating GC-B cells were 7AAD^-^ CD19^+^ CD3^-^ CFSE^neg^ and non-dividing GC-B cells were 7AAD^-^ CD19^+^ CD3^-^ CFSE^pos^. The gating strategy followed for re-sorting of these GC-Tfh and GC-B cells after co-culture is shown in **Fig 1D**.

#### Sorting of healthy human tonsillar mononuclear cells

GC-Tfh, pre-Tfh and autologous GC-B cells from healthy human tonsils were sorted as described above. After thawing the cells in complete RPMI, they were incubated with Benzonase for 30 minutes at 37°C then washed in medium and counted. Antibodies against human CD3, CD4, CXCR5, CD25, CD45RA, PD-1, CD19, CD319, IgD and CD38 were used to sort the cells. CD25 (BC96) and PD-1 (EH12.2H7) were purchased from Biolegend. Dead cells were excluded with the LIVE/DEAD fixable Aqua Dead Cell Stain Kit for flow cytometry (Vivid) (Life Technologies). After excluding doublets, sorted GC-Tfh cells were defined as Vivid^-^ CD3^+^ CD19^-^ CD4^+^ CD45RA^-^ CD25^-^ CXCR5^hi^ PD-1^hi^, pre-Tfh cells as Vivid^-^ CD3^+^ CD19^-^ CD4^+^ CD45RA^-^ CD25^-^ CXCR5^int^ PD-1^int^, and GC-B cells as Vivid^-^ CD19^+^ CD3^-^ CD38^int^ IgD^-^ CD319^-^. The gating strategy followed for the sorting is shown in **S3 Fig.**

#### Sorting of HIV^pos^ LN cells for RNA-seq

GC-Tfh and autologous GC-B cells were sorted from HIV^pos^ LN mononuclear cells as described above. Antibodies against human CD3, CD4, CXCR5, CD25, CD45RA, PD-1, CD19, CD319, IgD and CD38 were used and dead cells eliminated with Vivid. After excluding doublets, sorted GC-Tfh cells were Vivid^-^ CD3^+^ CD19^-^ CD4^+^ CD45RA^-^ CD25^-^ CXCR5^hi^ PD-1^hi^ and GC-B cells were Vivid^-^ CD19^+^ CD3^-^ CD38^int^ IgD^-^ CD319^-^. The gating strategy followed for sorting was similar to that used for healthy tonsillar cells shown in **S3 Fig**.

One day after co-culture of these sorted GC-Tfh cells from HIV^pos^ LNs with autologous GC-B cells, they were harvested and re-sorted for RNA-seq analysis. Antibodies against CD3, CD4 and CD19 were utilized. Dead cells were excluded using Vivid. After eliminating doublets, re-sorted GC-Tfh cells were Vivid^-^ CD3^+^ CD19^-^ CD4^+^. Sorted cells were directly collected in 75ul RNA lysis buffer (RLT (Qiagen) + 2-mercaptoethanol (ß-ME) (Sigma-Aldrich)) and snap-frozen on dry ice then stored at -80°C until RNA extraction was performed.

### Co-culture assays

#### LN CFSE^pos^ HIV^neg^ and HIV^pos^ GC-Tfh with autologous GC-B cells for gene array analysis

After sorting, GC-Tfh and GC-B cells were counted, washed and resuspended in complete RPMI at a density of 30,000 cells per a 10ul volume. Sorted CFSE^pos^ HIV^neg^ and HIV^pos^ LN GC-Tfh cells were plated in a 1:1 ratio with sorted autologous GC-B cells, in V-bottom shaped 96 well plates. The cells were co-cultured in presence of 100ng/ml of Staphylococcal Enterotoxin B (SEB) (Toxin Technology) in complete RPMI for a duration of 5 days before harvesting. The experimental layout and timeline leading to the gene array analysis is illustrated in **Fig 1B**.

#### TSL GC-Tfh or pre-Tfh cells with autologous GC-B cells for c-Maf and BATF intracellular expression analysis

Healthy TSL GC-Tfh, pre-Tfh and GC-B cells were handled and plated as the LN cells described above. GC-Tfh or pre-Tfh cells were co-cultured with autologous GC-B cells in presence of 100ng/ml of SEB with or without 10uM of the specific ADA-1 inhibitor (erythro-9-(2-hydroxy-3-nonyl)adenine) (EHNA) (Tocris) in complete RPMI. Cells were harvested on day 1, while supernatants were harvested on day 5 after co-culture.

#### LN HIV^pos^ GC-Tfh with autologous GC-B cells for RNA-seq analysis

After sorting, GC-Tfh and GC-B cells were handled as described above but plated in complete RPMI at a density of 20,000 cells per a 10ul volume. The cells were co-cultured in presence of 100ng/ml of SEB with or without 2.4uM ADA-1 (Sigma-Aldrich) in complete RPMI. Cells were harvested 1 day after co-culture.

### Intracellular flow cytometric analysis

GC-Tfh, pre-Tfh and autologous GC-B cells from healthy human TSLs were harvested 1 day after co-culture and GC-Tfh as well as pre-Tfh cells analyzed by flow cytometry for their intracellular expression of c-Maf and the upstream transcription factor of its signaling pathway BATF. Harvested cells were resuspended in Vivid for 10 minutes to exclude dead cells, before washing and adding fluorochrome-conjugated anti-human antibodies against surface CD4 and CD45RA in FACS buffer. After a 20-minute incubation on ice, cells were washed then fixed for 1 hour at 4°C away from the light, using Foxp3 fixation/permeabilization buffer (eBioscience). Fluorochrome-conjugated anti-human antibodies against intracellular c-Maf (sym0F1) and BATF (MBM7C7) were then added to the cells in 1X permeabilization buffer (eBioscience) and incubated for 30 minutes to 1 hour at 4°C in the dark. For staining negative controls labeled as Fluorescence Minus One (FMO), cells were incubated with 1X permeabilization buffer alone. Cells were resuspended in PBS + 2% FBS after 2 washing steps and were acquired on a BD LSR II (BD Biosciences) and analyzed with FlowJo software (Treestar). After doublet exclusion, Vivid^-^ CD4^+^ CD45RA^-^ GC-Tfh and pre-Tfh cells were analyzed separately for their high intracellular expression of c-Maf (c-Maf^hi^) and BATF (BATF^hi^). The cell gating strategy followed is shown in **S1 Fig**.

### Total IgG quantification by ELISA

Total IgG was measured in supernatants on day 5 following co-culture as previously described [43]. Coating of 96-well Immunlon 2HB ELISA plates (Thermo Scientific) was done overnight at 4°C using monoclonal anti-human IgG antibody (Clone MT91/145; Mabtech) at a concentration of 1ug/ml in PBS. The following day, plates were washed 4 times with wash buffer (PBS + 0.05% Tween-20) and incubated for 1 hour at room temperature (RT) with PBS + 10% FBS. Standards, blanks and samples were loaded onto appropriate plate wells after another washing step, and were left to incubate for 1 hour at RT. Standards were added in duplicates in a 2-fold serial dilution. After sample incubation and washing, anti-human IgG-biotin antibody (Clone MT78/145; Mabtech) was added at a concentration of 1ug/ml in PBS + 10% FBS and plates incubated at RT for 1 hour before another washing step and the addition of streptavidin-HRP (Mabtech) for 1 hour at RT. Next, plates were washed 5 times before the TMB substrate (Sigma-Aldrich) was added to all wells until a blue color was observed. The reaction was then stopped with 1M H_3_PO_4_. Absorbance was read at 450nm with the use of a SpectraMax plus 384 plate reader (Molecular Devices) for assays pertaining to the gene array experiments, and a Synergy HTX multi-mode (BioTek) spectrophotometer for assays pertaining to co-cultures from healthy human TSLs. Readings were made within 15 minutes of stopping the reaction. Standard curves were generated and sample concentrations were calculated in ng/ml.

### Cytokine and chemokine quantification by Luminex

The ProcartaPlex Multiplex Immunoassay (28-Plex) (ThermoFisher Scientific) was used for the detection of 28 human cytokines and chemokines produced by healthy tonsillar GC-Tfh and pre-Tfh cells in co-culture with GC-B cells. Supernatants were collected on day 5 after co-culture and the following human cytokine/chemokine premixed panel was used according to the manufacturer’s protocol: BLC, CD40L, Fractalkine, GM-CSF, IFNα, IFNγ, IL-1ß, IL-10, IL-12p70, IL-13, IL-15, IL-17A, IL-2, IL-21, IL-22, IL-23, IL-4, IL-6, IL-8, IL-9, IP-10, MCP-1, MIP-1α, MIP-1ß, MIP-3α, SDF-1α, TNFα, TSLP. Briefly, 50ul of the magnetic beads were vortexed and added to each well of the assay plate, then removed by plate washing. Then, 25ul of the 1X Universal Assay buffer were added to all wells, followed by 50ul of prepared standards, blanks and samples into appropriate wells. Standards were run in duplicates in a 4-fold serial dilution. Culture medium was used as blank. Plates were sealed and incubated for 2 hours. Next, a washing step preceded the addition of 25ul of 1X detection antibody mixture to all wells and incubation for 30 minutes. Following another wash, 50ul of streptavidin-PE were dispensed in all wells and plates were incubated for another 30 minutes. Lastly, plates were washed again before the addition of 120ul of reading buffer followed by a 5-minute incubation. All incubations were done on a plate shaker (at 500 rpm) at RT, away from the light. Data was acquired on a Bio-Plex 200 System using beads regions defined in the protocol and analyzed with the Bio-Plex Manager 6.1 software (Bio-Rad). Standard curves were generated and sample concentrations were calculated in pg/ml.

### RNA extraction and microarray of CFSE^neg^ and CFSE^pos^ LN HIV^neg^ and HIV^pos^ GC-Tfh cells

Five days after co-culture, CFSE^neg^ and CFSE^pos^ LN GC-Tfh cells from HIV-infected and -uninfected individuals were re-sorted into 100ul of cold RLT buffer (Qiagen) supplemented with 1% βM (Sigma Aldrich), and quickly stored at −80°C. Extraction of total RNA followed by DNase I treatment was performed using Qiagen’s RNeasy Micro Kit according to the manufacturer’s protocol. RNA was quantified with a NanoDrop spectrophotometer (Thermo Scientific) and its quality measured using the Experion automated electrophoresis system (Bio-Rad) along with a HeLa RNA positive control and a non-template negative control. RNA was converted into biotinylated cRNA using the Illumina Total Prep-96 RNA amplification kit (Life Technologies). Biotinylated cRNA was normalized and hybridized to the Illumina Human HT-12V4 Expression BeadChips according to the manufacturer’s guidelines, then quantified with an Illumina iScan system (Illumina). Data was collected using Illumina GenomeStudio software.

### RNA extraction and RNA-seq of LN HIV^pos^ GC-Tfh cells

After 1 day in co-culture with autologous GC-B cells, LN HIV^pos^ GC-Tfh cells were directly sorted into cold RLT buffer (Qiagen) supplemented with 1% βM (Sigma Aldrich), snap-frozen on dry ice then quickly stored at −80°C. Total RNA was isolated using the RNeasy Micro Kit (Qiagen) following recommended procedures, with on-column DNase I treatment. Total RNA was normalized prior to oligo-dT capture and cDNA synthesis with SMART-Seq v4 (Takara). RNA libraries were generated using the Nextera XT DNA Library Prep Kit (Illumina). All sample quality assessment was performed on a 5300 Fragment Analyzer System (Agilent) and quantified using a Qubit 3.0 fluorometer (Life Technologies). Medium depth sequencing (>16 million reads per sample) was performed on a NextSeq 550 System (Illumina) using two High Output flow cells each with a 75-base pair, Paired End run.

Demultiplexed fast-q paired end read adapters of length less than 36 and average phred quality score of less than 30 were trimmed and filtered using the skewer software [107]. Alignment was performed with HISAT2 to the Homo sapiens NCBI reference genome assembly version GRCh38 and sorted with SAMtools [108,109]. The aligned reads were counted and assigned gene meta-information using the featureCounts software [110].

### Microarray and RNA-seq analysis of LN GC-Tfh cells

The microarray and RNA-seq expression analysis was conducted using the R programming language and LIMMA from the Bioconductor suite [111]. RNA-seq transcripts were first filtered based on the limit of detection, TMM-normalized, and transformed to fulfill modeling assumptions. Likewise, the microarray data was first background-corrected, quantile-normalized, and transformed. Both preprocessed data sets were then assessed for normality and uniformity between samples prior to analysis, where a single CFSE^pos^ HIV^pos^ sample was removed due to a substantially non-normal expression distribution, and a single CFSE^neg^ HIV^neg^ sample was removed because it was noted below detection level in the microarray analysis. RNA-seq samples were then voom-transformed to correct for heteroscedasticity, and analysis of both microarray and RNA-seq was performed using moderated t-tests with an empirical Bayesian adjustment. Functional gene set enrichment analysis was performed using the Gene Set Variation Analysis (GSVA) library and gene sets as defined by Ingenuity Pathway Analysis (IPA) and Molecular Signatures Databases (MSigDB) [112,113]. Nominal p-values of p<0.05 (marked in the text and figures as p<0.05, p<0.01, p<0.001 and p<0.0001, and additionally in Fig 8 as *p<0.05) were considered significant. Where indicated, False Discovery Rate (FDR) or Benjamini Hochberg adjusted p-value of <0.05 was used.

### Statistical analyses

For non-bioinformatics data, Prism 6 (GraphPad software) was used for analysis. Results were represented as Means and Standard Errors (Mean ± SEM). Statistical significance was determined with the unpaired two-tailed non-parametric Student’s t-test using the Mann-Whitney test or the two-tailed paired non-parametric Student’s t-test using the Wilcoxon matched-pairs signed rank test. One-way ANOVA followed by the Tukey multiple comparisons test was used where appropriate. Nominal p-values of p<0.05 (marked in the figures as * p<0.05, ** p<0.01 and *** p<0.001) were considered significant.

## Supporting information

S1 Fig Flow cytometric gating strategy for c-Maf and BATF expression in healthy tonsillar GC-Tfh and pre-Tfh cells from co-cultures with GC-B cells. Sorted human healthy tonsillar GC-Tfh (live CD3^+^ CD4^+^ CD45RA^-^ CD25^-^ CXCR5^hi^ PD-1^hi^) and pre-Tfh (live CD3^+^ CD4^+^ CD45RA^-^ CD25^-^ CXCR5^int^ PD-1^int^) cells were co-cultured with autologous GC-B cells (live CD19^+^ CD38^int^ IgD^-^ CD319^-^) in a 1:1 ratio in presence of SEB (SEB) with or without 10uM of the ADA-1 specific inhibitor EHNA (EHNA). Cells were harvested on day 1 after co-culture and stained intracellularly for high expression of c-Maf and BATF by flow cytometry. After doublet and dead cell exclusion, GC-Tfh and pre-Tfh cells were gated as CD4^+^ CD45RA^-^ c-Maf^hi^ BATF^hi^ for measurement of c-Maf and BATF expression.
(TIF)

S2 Fig The specific ADA-1 inhibitor, EHNA, does not compromise GC-Tfh and pre-Tfh cell viability. Sorted human healthy tonsillar GC-Tfh (live CD3^+^ CD4^+^ CD45RA^-^ CD25^-^ CXCR5^hi^ PD-1^hi^) and pre-Tfh (live CD3^+^ CD4^+^ CD45RA^-^ CD25^-^ CXCR5^int^ PD-1^int^) cells were co-cultured with autologous GC-B cells (live CD19^+^ CD38^int^ IgD^-^ CD319^-^) in a 1:1 ratio in presence of SEB (SEB) with or without 10uM of the ADA-1 specific inhibitor EHNA (EHNA). Cells were harvested on day 1 after co-culture and stained for viability with LIVE/DEAD fixable Aqua Dead Cell Stain Kit for flow cytometry (Vivid). Live cells were gated as Vivid-negative after doublet cell exclusion as in **S1 Fig**. Results are from 3 independent experiments (n = 8–9) and are represented as mean ± SEM. Data was analyzed with the two-tailed paired non-parametric Student’s t-test using the Wilcoxon matched-pairs signed rank test. Nominal p-values p<0.05 were considered of statistical significance.
(TIF)

S3 Fig Flow cytometric gating strategy for the sorting of healthy tonsillar and HIV^pos^ LN cells. Human healthy tonsillar GC-Tfh, pre-Tfh and autologous GC-B cells were sorted on day 0, before plating in co-culture to assess T cell intracellular c-Maf and BATF expression. After doublet exclusion, sorted GC-Tfh cells were defined as Vivid^-^ CD3^+^ CD19^-^ CD4^+^ CD45RA^-^ CD25^-^ CXCR5^hi^ PD-1^hi^, pre-Tfh cells as Vivid^-^ CD3^+^ CD19^-^ CD4^+^ CD45RA^-^ CD25^-^ CXCR5^int^ PD-1^int^, and GC-B cells as Vivid^-^ CD19^+^ CD3^-^ CD38^int^ IgD^-^ CD319^-^. Similarly, HIV^pos^ LN GC-Tfh and autologous GC-B cells used for RNA-seq analysis, were sorted on day 0 following the same gating strategy, before plating in co-culture.
(TIF)

S4 Fig Proposed model for the impairement of GC-Tfh/GC-B cell interaction during chronic HIV infection. In chronic HIV infection, the virus alters the LN GC reaction by impairing the interaction of GC-Tfh with GC-B cells, leading to an inadequate anti-HIV humoral response. The virus downregulates ADA-1 expression, which disrupts the cytokine balance, namely the low IL-2/IL-6 ratio, crucial for the proper GC-Tfh function in B cell help. IL-6 downregulation attenuates IL-6 signaling via the IL-6R, consequently reducing the c-Maf pathway activation, by decreasing STAT3, BATF and ultimately c-Maf expression. In addition, the upregulation and engagement of IL-2 with its receptor may attenuate c-Maf through STAT5 activation. HIV also triggers PD-1/PD-L1 interaction on GC-Tfh and GC-B cells respectively. PD-L1-induced signaling through defective HIV^pos^ GC-B cells, may also inhibit c-Maf activation. This Figure was created with BioRender.com.
(TIF)

S1 Table Differential gene expression in proliferating GC-Tfh cells from HIV-infected and HIV-uninfected LNs. We used single-gene analysis to determine the pattern of gene expression in proliferating LN GC-Tfh cells in co-culture with autologous GC-B cells from HIV^pos^ patients versus HIV^neg^ subjects. We generated heatmaps of the top 100 DEGs in proliferating (CFSE^neg^ vs CFSE^pos^) HIV^neg^ and HIV^pos^ GC-Tfh cells compared to non-dividing cells (**Fig 3B and 3C**). This Table comprises all top 100 DEGs shown in the heatmaps with their log2-fold changes (logFC) as well as nominal p-values. The first sheet displays the top upregulated and downregulated DEGs in proliferating HIV^neg^ GC-Tfh cells, whereas the second sheet displays the top upregulated and downregulated DEGs in proliferating HIV^pos^ GC-Tfh cells. The included DEGs have the highest positive or negative logFC and statistically significant nominal p-values of <0.05.
(XLSX)

S2 Table Differential pathway expression analysis in proliferating HIV^neg^ and HIV^pos^ GC-Tfh cells. We used Gene Set Variation Analysis (GSVA) to determine the biological characterization, statistical significance and differences in selected databases. We performed enrichment analyses using Ingenuity Pathway Analysis (IPA) gene sets to determine the profile of GC-Tfh cell proliferation in the context of HIV or a non-HIV environment. We generated checkerboard plots representing the top lists of enriched pathways in the DEGs specific to proliferating GC-Tfh cells in the HIV^neg^ and HIV^pos^ contexts (**Fig 4A and 4B**). The top enriched pathway list in HIV^neg^ cells is shown in the first Table sheet while the top enriched pathway list in the HIV^pos^ cells is shown in the second Table sheet. Pathways are listed with their log2-fold change (logFC) and their nominal p-value. Statistical significance was considered with nominal p-values p<0.05.
(XLSX)

S3 Table Venn diagram analysis of proliferating HIV^neg^ and HIV^pos^ GC-Tfh cells. We generated Venn diagrams to show the numbers of unique and common statistically significant DEGs in proliferating HIV^neg^ and HIV^pos^ GC-Tfh cell populations (**Fig 5C and 5D**). This Table shows the lists of upregulated (first sheet) and downregulated (second sheet) DEGs, that are unique as well as common to HIV^neg^ and HIV^pos^ proliferating (CFSE^neg^ vs CFSE^pos^) GC-Tfh cells. Analysis was performed based on nominal p-value p<0.05.
(XLSX)

S4 Table RNA-seq analysis of LN HIV^pos^ GC-Tfh cells after co-culture with ADA-1 supplementation. We analyzed with RNA-seq GC-Tfh cells treated in co-culture with SEB, in presence or absence of ADA-1. We used Gene Set Variation Analysis (GSVA) by examining Molecular Signatures Databases (MSigDB) Canonical Pathways. We generated heatmaps illustrating the differential gene expression in the IL-6, IL-2 and Th1/Th2 pathways in HIV^pos^ GC-Tfh cells treated in co-culture with ADA-1 versus SEB alone (**Fig 8**). This Table shows all the upregulated and downregulated DEGs listed in the IL-6 (first sheet), IL-2 (second sheet) and Th1/Th2 (third sheet) pathways, along with their positive or negative log2-fold change (logFC) as well as nominal p-values. Statistical significance was considered with p<0.05.
(XLSX)
