# Supplementary figures and images for: Germinal Center T follicular helper (GC-Tfh) cell impairment in chronic HIV infection involves c-Maf signaling
Source: PLoS Pathog. 2021 Jul 19;17(7):e1009732. doi: 10.1371/journal.ppat.1009732 (PMC8289045; doi:10.1371/journal.ppat.1009732)

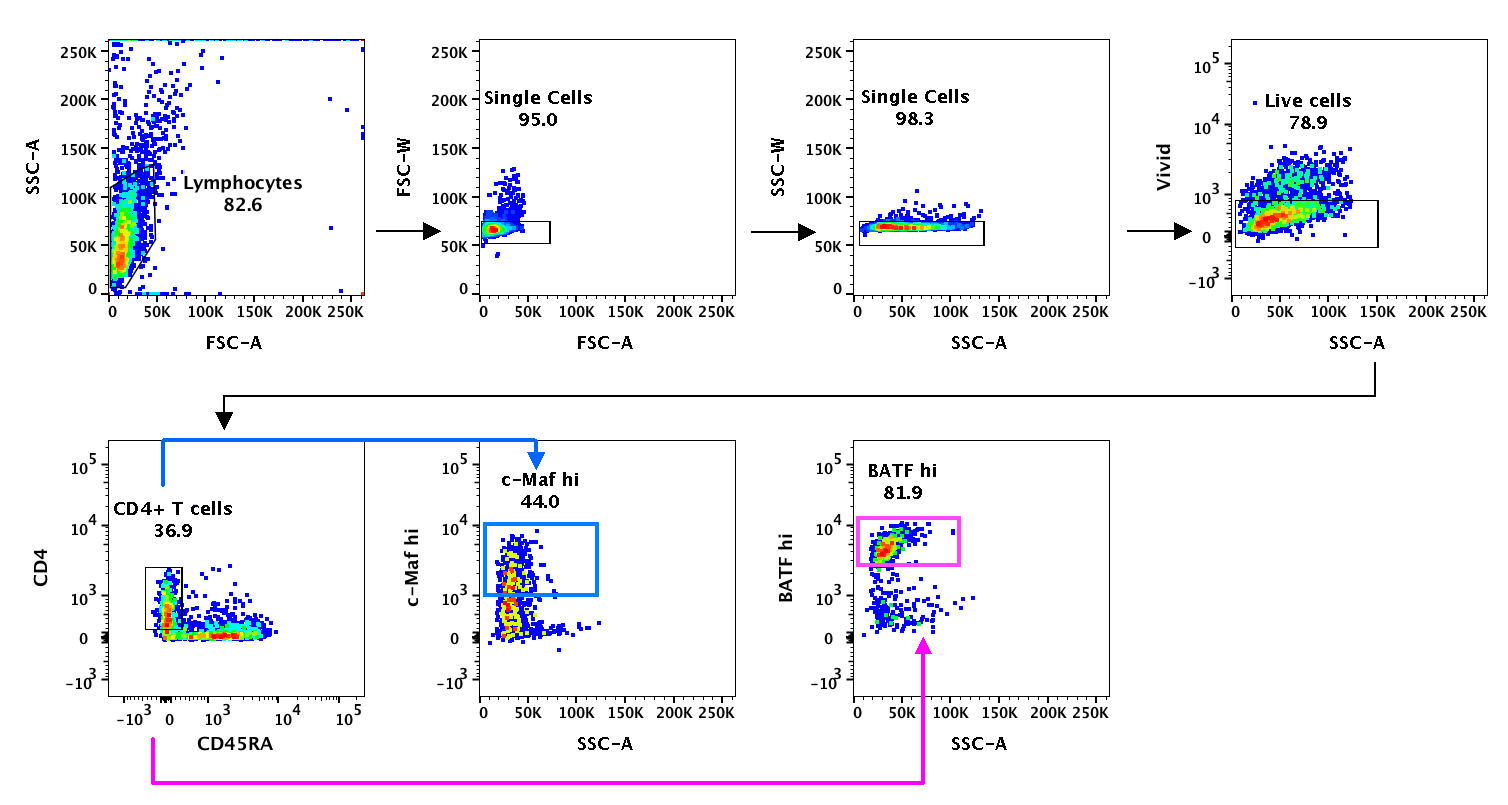

Supplement: S1 Fig — Sorted human healthy tonsillar GC-Tfh (live CD3+ CD4+ CD45RA- CD25- CXCR5hi PD-1hi) and pre-Tfh (live CD3+ CD4+ CD45RA- CD25- CXCR5int PD-1int) cells were co-cultured with autologous GC-B cells (live CD19+ CD38int IgD- CD319-) in a 1:1 ratio in presence of SEB (SEB) with or without 10uM of the ADA-1 specific inhibitor EHNA (EHNA). Cells were harvested on day 1 after co-culture and stained intracellularly for high expression of c-Maf and BATF by flow cytometry. After doublet and dead cell exclusion, GC-Tfh and pre-Tfh cells were gated as CD4+ CD45RA- c-Mafhi BATFhi for measurement of c-Maf and BATF expression. (TIF) [file ppat.1009732.s001.tif]

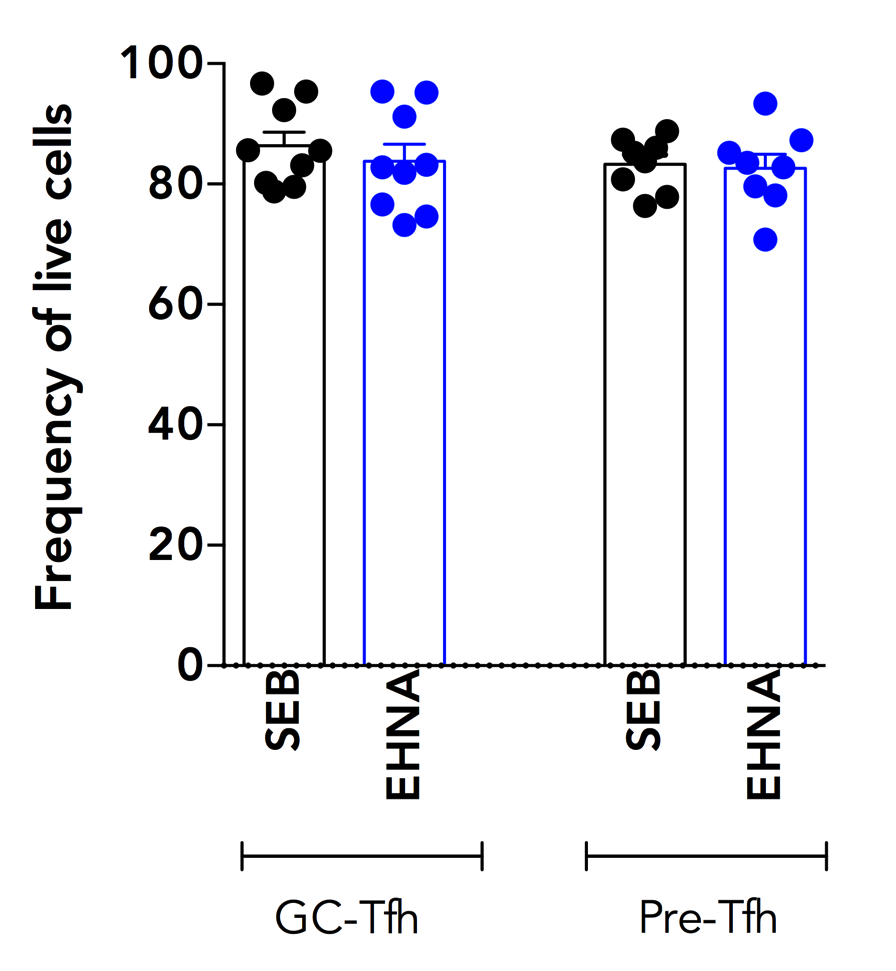

Supplement: S2 Fig — Sorted human healthy tonsillar GC-Tfh (live CD3+ CD4+ CD45RA- CD25- CXCR5hi PD-1hi) and pre-Tfh (live CD3+ CD4+ CD45RA- CD25- CXCR5int PD-1int) cells were co-cultured with autologous GC-B cells (live CD19+ CD38int IgD- CD319-) in a 1:1 ratio in presence of SEB (SEB) with or without 10uM of the ADA-1 specific inhibitor EHNA (EHNA). Cells were harvested on day 1 after co-culture and stained for viability with LIVE/DEAD fixable Aqua Dead Cell Stain Kit for flow cytometry (Vivid). Live cells were gated as Vivid-negative after doublet cell exclusion as in S1 Fig. Results are from 3 independent experiments (n = 8–9) and are represented as mean ± SEM. Data was analyzed with the two-tailed paired non-parametric Student’s t-test using the Wilcoxon matched-pairs signed rank test. Nominal p-values p<0.05 were considered of statistical significance. (TIF) [file ppat.1009732.s002.tif]

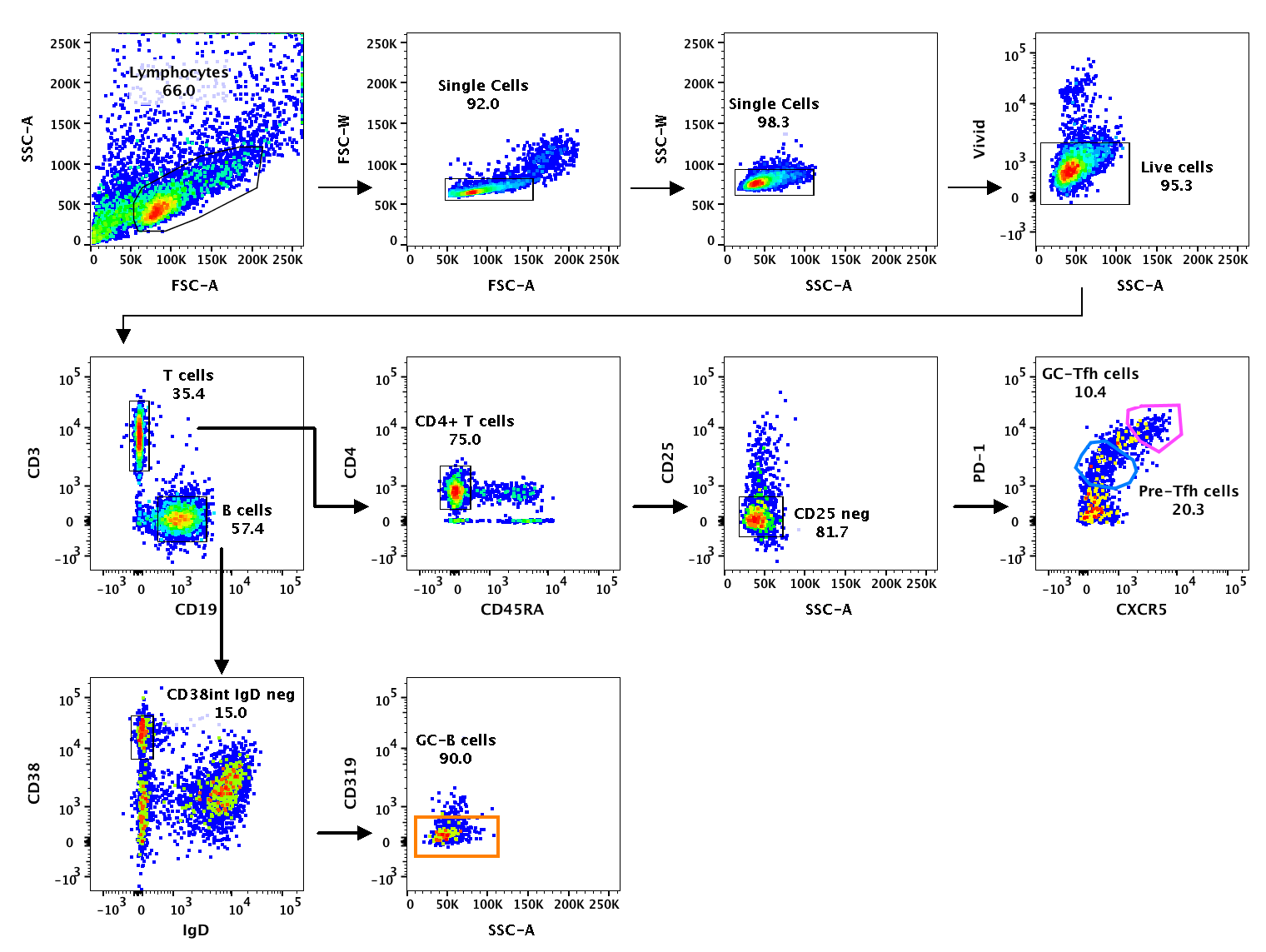

Supplement: S3 Fig — Human healthy tonsillar GC-Tfh, pre-Tfh and autologous GC-B cells were sorted on day 0, before plating in co-culture to assess T cell intracellular c-Maf and BATF expression. After doublet exclusion, sorted GC-Tfh cells were defined as Vivid- CD3+ CD19- CD4+ CD45RA- CD25- CXCR5hi PD-1hi, pre-Tfh cells as Vivid- CD3+ CD19- CD4+ CD45RA- CD25- CXCR5int PD-1int, and GC-B cells as Vivid- CD19+ CD3- CD38int IgD- CD319-. Similarly, HIVpos LN GC-Tfh and autologous GC-B cells used for RNA-seq analysis, were sorted on day 0 following the same gating strategy, before plating in co-culture. (TIF) [file ppat.1009732.s003.tif]

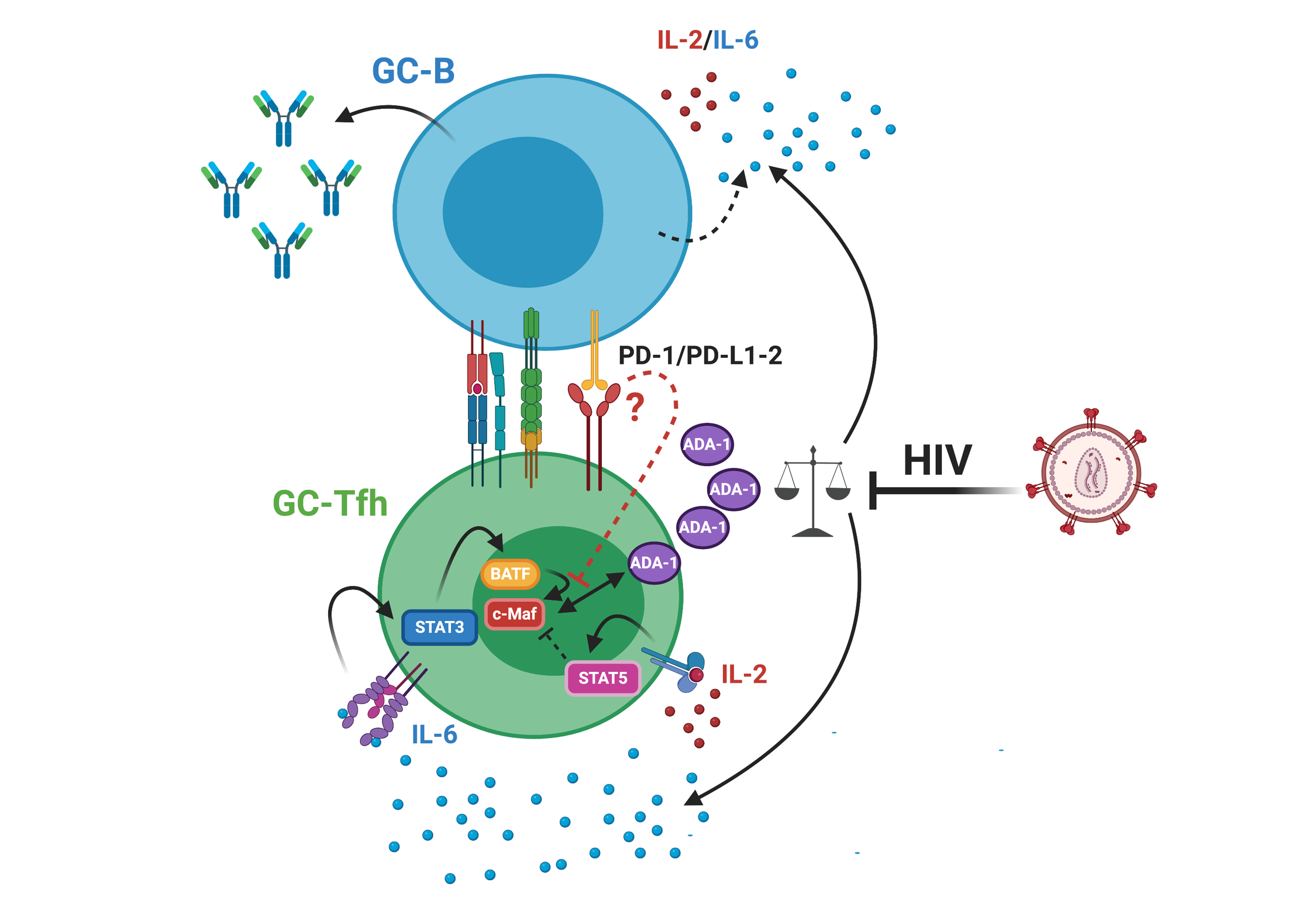

Supplement: S4 Fig — In chronic HIV infection, the virus alters the LN GC reaction by impairing the interaction of GC-Tfh with GC-B cells, leading to an inadequate anti-HIV humoral response. The virus downregulates ADA-1 expression, which disrupts the cytokine balance, namely the low IL-2/IL-6 ratio, crucial for the proper GC-Tfh function in B cell help. IL-6 downregulation attenuates IL-6 signaling via the IL-6R, consequently reducing the c-Maf pathway activation, by decreasing STAT3, BATF and ultimately c-Maf expression. In addition, the upregulation and engagement of IL-2 with its receptor may attenuate c-Maf through STAT5 activation. HIV also triggers PD-1/PD-L1 interaction on GC-Tfh and GC-B cells respectively. PD-L1-induced signaling through defective HIVpos GC-B cells, may also inhibit c-Maf activation. This Figure was created with BioRender.com. (TIF) [file ppat.1009732.s004.tif]
